# Crystal size, shape, and conformational changes drive both the disappearance and reappearance of ritonavir polymorphs in the mill

**DOI:** 10.1073/pnas.2319127121

**Published:** 2024-04-01

**Authors:** Pietro Sacchi, Sarah E. Wright, Petros Neoptolemou, Giulio I. Lampronti, Ashwin Kumar Rajagopalan, Weronika Kras, Caitlin L. Evans, Paul Hodgkinson, Aurora J. Cruz-Cabeza

**Affiliations:** ^a^Department of Chemical Engineering, University of Manchester, Manchester M13 9PL, United Kingdom; ^b^The Cambridge Crystallographic Data Centre, Cambridge CB2 1EZ, United Kingdom; ^c^Department of Earth Sciences, University of Cambridge, Cambridge CB2 3EQ, United Kingdom; ^d^Chemical Development, Pharmaceutical Technology & Development, AstraZeneca, Macclesfield SK10 2NA, United Kingdom; ^e^Department of Chemistry, Durham University, Durham DH1 3LE, United Kingdom

**Keywords:** crystal polymorphism, pharmaceuticals, particle size and shape, mechanochemistry

## Abstract

Organic compounds can crystallize in different forms known as polymorphs. Discovery and control of polymorphism is crucial to the pharmaceutical industry since different polymorphs can have significantly different physical properties which impacts their utilization in drug delivery. Certain polymorphs have been reported to ‘disappear’ from the physical world, irreversibly converting to new ones. These unwanted polymorph conversions, initially prevented by slow nucleation kinetics, are eventually observed driven by significant gains in thermodynamic stabilities. The most infamous of these cases is that of the HIV drug ritonavir (RVR): Once its reluctant form was unwillingly nucleated for the first time, its desired form could no longer be produced with the same manufacturing process. Here we show that RVR’s extraordinary disappearing polymorph as well as its reluctant form can be consistently produced by ball-milling under different environmental conditions. We demonstrate that the significant difference in stability between its polymorphs can be changed and reversed in the mill—a process we show is driven by crystal size as well as crystal shape and conformational effects. We also show that those effects can be controlled through careful design of milling conditions since they dictate the kinetics of crystal breakage, dissolution, and growth processes that eventually lead to steady-state crystal sizes and shapes in the mill. This work highlights the huge potential of mechanochemistry in polymorph discovery of forms initially difficult to nucleate, recovery of disappearing polymorphs, and polymorph control of complex flexible drug compounds such as RVR.

## Significance

Late-appearing polymorphism in pharmaceuticals can have devastating consequences for drug delivery. We show, on the infamous drug Ritonavir (RVR), how ball mill grinding experiments under carefully designed conditions (solvent, milling times…) allow for the easy discovery of its reluctant polymorph (form II) as well as the recovery of its disappearing polymorph (form I). The thermodynamic stability of these polymorphs is reversed in the mill, and this is shown (with the aid of molecular simulations) to be a consequence of not only crystal size but also crystal shape and molecular conformation effects. Carefully designed ball milling offers unprecedented control over conformational polymorphism (exemplified with RVR), having the potential to reshape pharmaceutical solid form discovery and development.

Tablets are the most common vehicles for drug delivery. They contain powders of active pharmaceutical ingredients (APIs) together with excipients. Although pharmaceutical solids are generally stable and easy to handle, they also face multiple challenges in their journey through pharmaceutical development. Perhaps one of the most important challenge of them all is that of crystal polymorphism, (1) especially when it is late appearing, (2) disappearing (3, 4), or uncontrolled (5). Polymorphism is the ability of a compound to crystallize in more than one crystal structure (1) often also involving significant changes in molecular conformation (conformational polymorphs) (6). With the ever-increasing molecular complexity of modern APIs, controlling the impact of molecular flexibility in polymorphism (6) and crystallization (7, 8) is critical to enable the development of modern drugs.

The most infamous example of conformational and disappearing polymorphism is undoubtably that of ritonavir (RVR) (2). RVR, an HIV-protease inhibitor antiviral for treating acquired immune deficiency syndrome, was developed and commercialized by Abbot Laboratories in the form of gel capsules formulated based on the knowledge of RVR form I (RVR-I). Two years after market launch, the manufacturing process designed to produce RVR-I yielded form II (RVR-II), compromising the product’s solubility and bioavailability (2). Intriguingly, once RVR-II crystal seeds had formed at any of Abbot’s laboratories or manufacturing plants, RVR-I could not be obtained any longer. The capsules had to be withdrawn from the market and reformulated after significant efforts from Abbot’s scientists (9). The “disappearance” of a metastable polymorph during crystallization occurs the moment a significantly more stable (but previously unknown) polymorph is inadvertently nucleated. This process—initially prevented kinetically by the difficulty of nucleating the stable form—is driven by the significant thermodynamic stability difference between the polymorphs. Once nuclei of the stable form exist, however, the kinetic barriers for nucleation disappear, and the stable form (for RVR its form II) becomes dominant under most crystallization conditions.

**Fig. 1.**
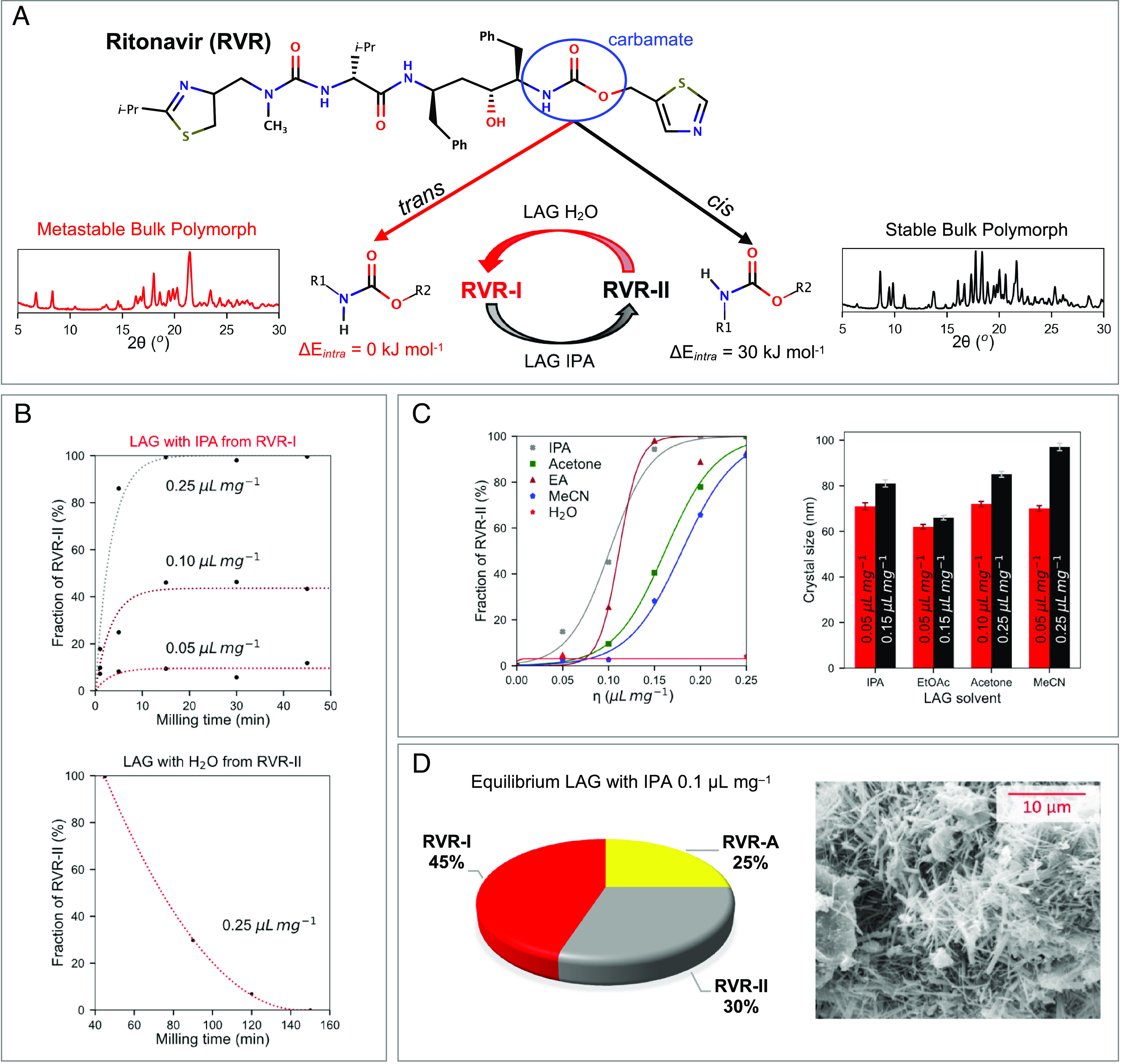
(*A*) RVR molecular structure, carbamate configurations, and RVR-I and RVR-II interconversion upon milling. (*B*) Kinetics of the RVR-I to RVR-II conversion (LAG with IPA, *Upper*) and the RVR-II to RVR-I conversion (LAG with water, *Lower*). (*C*) Equilibrium milling curves as a function of solvent concentration (*η*) in five solvents (*Left*, EA = ethylacetate and MeCN = acetonitrile) and crystal sizes from PXRD data analysis around for RVR-I and RVR-II around the critical solvent concentration. (*D*) Phase composition of the equilibrium product of RVR LAG with IPA at 0.10 μL mg^−1^ (*Left*) with SEM image (*Right*). The lines in the plots serve as a guide for the eye. In the plot of panel *C*, lines were obtained by fitting a sigmoid model. Form abbreviations are RVR-I (for RVR-I), RVR-II (for RVR-II), and RVR-A (for amorphous RVR).

The RVR molecule is large and flexible with the configuration around several of its rotatable bonds changing in its two polymorphs (*SI Appendix*, Figs. S1 and S2). For simplicity and because of its significance, we focus our attention on the carbamate group, which adopts a stable *trans* configuration in RVR-I and a metastable (+30 kJ mol^−1^) *cis* configuration in RVR-II (Fig. 1*A*). The *cis* configuration is not only disfavored thermodynamically but also kinetically because of the very high energy barrier for the *trans*-to-*cis* interconversion (~90 kJ mol^−1^) (10). The difficulties of nucleating RVR-II have partly been ascribed to a low abundance of the *cis* conformer in solution (2), but what would happen if we were not to use solution crystallization?

## Results and Discussion

Here we study the polymorphism of RVR by means of neat (NG) and liquid-assisted (LAG) ball-mill grinding (11). First, we explore whether the conversion between RVR-I and RVR-II, with the required conformational changes, is possible by milling. It has been previously shown that the environmental conditions of ball-milling experiments (i.e., the amount of solvent or its nature) (11–14) are instrumental for enabling polymorphic transformations in the mill (15). After some experimental exploration, conditions were found for which RVR-I and RVR-II could consistently interconvert in the mill with conversion kinetics shown in Fig. 1*B* for LAG experiments of RVR with isopropanol (IPA) and water. From RVR-I, a full conversion to RVR-II was achieved after just a few minutes by milling with IPA at 0.25 µL mg^−1^. This observation is remarkable, considering the reported difficulty of nucleating crystals of RVR-II from solution (2). From RVR-II, LAG with water enabled conversion to RVR-I, but it required significantly longer milling times (>120 min). It is to be highlighted that establishing the required milling environmental conditions as well as the required milling times to achieve steady state requires careful initial exploration [and establishing of appropriate induction times (16, 17)]; otherwise, important form conversions will not be observed and milling will not lead to stable forms (18).

Once conversion kinetics were established with IPA, milling equilibrium curves were produced for five different solvents (Fig. 1*C*). In these curves, the phase composition of the LAG product at steady state is shown as a function of solvent concentration. Milling with no solvent (neat-grinding, NG, or at low solvent concentrations) always afforded RVR-I. In all tested solvents except water, as the solvent concentration was increased, RVR-II started appearing until it became the sole phase at concentrations above ~0.15 to 0.25 µL mg^−1^. The shallow slopes of the equilibrium curves suggest the presence of an additional third phase in the milling products, in accordance with previous observations (13, 19) and with the theory of cooperativity of Hunter and Anderson (20). As new diffraction peaks in the PXRD (powder X-ray diffraction) patterns of the products were absent, the third phase must be constituted by amorphous RVR (RVR-A). This is expected since milling can lead to amorphization (21) and all our milling experiments were performed at room temperature, which is significantly below the glass transition temperature of RVR-A (T_g_ = 50 °C) (22).

Amorphous phase quantification of the LAG product with IPA (0.10 µL mg^−1^) was performed via Rietveld analysis with the internal standard method. Under those conditions, the milling powders were composed of ~45% RVR-I, ~30% RVR-II, and ~25% of RVR-A (Fig. 1*D*). An SEM (scanning electron microscopy) image of the milling product showed regions with aggregated particles and regions with dispersed needles. Full amorphization of RVR was achieved upon NG for over 120 min. RVR-A was produced from both RVR-I and RVR-II and two independent spectroscopic methods confirmed that both RVR-A phases obtained were identical [^13^C CP/MAS (cross-polarization/magic-angle spinning) and Fourier Transform Infrared Spectroscopy (FTIR), *SI Appendix*, Figs. S18 and S19 respectively]. The FTIR spectra of RVR-I, RVR-II, and RVR-A revealed a greater structural similarity of RVR-A with RVR-I suggesting that the more stable *trans* carbamate conformation is also dominant in RVR-A (*SI Appendix*, Fig. S19). The crystallite sizes (Scherrer) of the LAG products in various solvents, calculated for the minimum solvent concentration affording at least 85% of each phase, were found to have average values of 68.9 ± 1.3 nm and 82.5 ± 1.4 nm for RVR-I and RVR-II, respectively (Fig. 1*C*). Smaller crystallite sizes were found for milling conditions that lead to RVR-I (lower solvent concentration). We note that crystallite sizes for the products in ethyl acetate are particularly small (~60 nm) for both polymorphs, suggesting that crystal surfaces might be particularly stabilized in this solvent.

Since polymorphs typically differ in lattice energies by just a few kJ mol^−1^, (23–25) differences in their surface structures and stabilities can lead to thermodynamic stability switches at the nanoscale (13, 25). This has been shown to drive polymorphic conversions in the mill for relatively simple molecules (13, 26, 27). The energy differences for conformational polymorphs, however, are more significant than packing polymorphs (23), and we found surprising to observe similar conversions for RVR.

To explore the thermodynamics of the RVR polymorphs at the nanoscale, we performed state-of-the-art DFT-d calculations of the particle energies of RVR-I and RVR-II as a function of size and shape using Eq. **1** (13).

[1] $$\begin{array}{c} E_{\mathrm{particle}}\left(r,\;\phi \right)=\left(E_{latt-inter}+\Delta E_{\mathrm{intra}}\right)\\ \;+(-0.5\sum_{\left(hkl\right)}x_{\left(hkl\right)}\;(r,\;\phi )\;E_{\mathrm{att}}^{\left(hkl\right)}). \end{array}$$

The first term in the equation is the lattice energy (with an inter- and an intramolecular part) and the second term is the surface energy penalty of the particle which can be computed by adding up the individual surface energies of the (hkl) facets weighted by their morphological importance, $x_{\left(hkl\right)}\left(r,\;\phi \right)$   . The morphological importance depends on the size, *r*, and shape, ϕ , of the crystal.

**Fig. 2.**
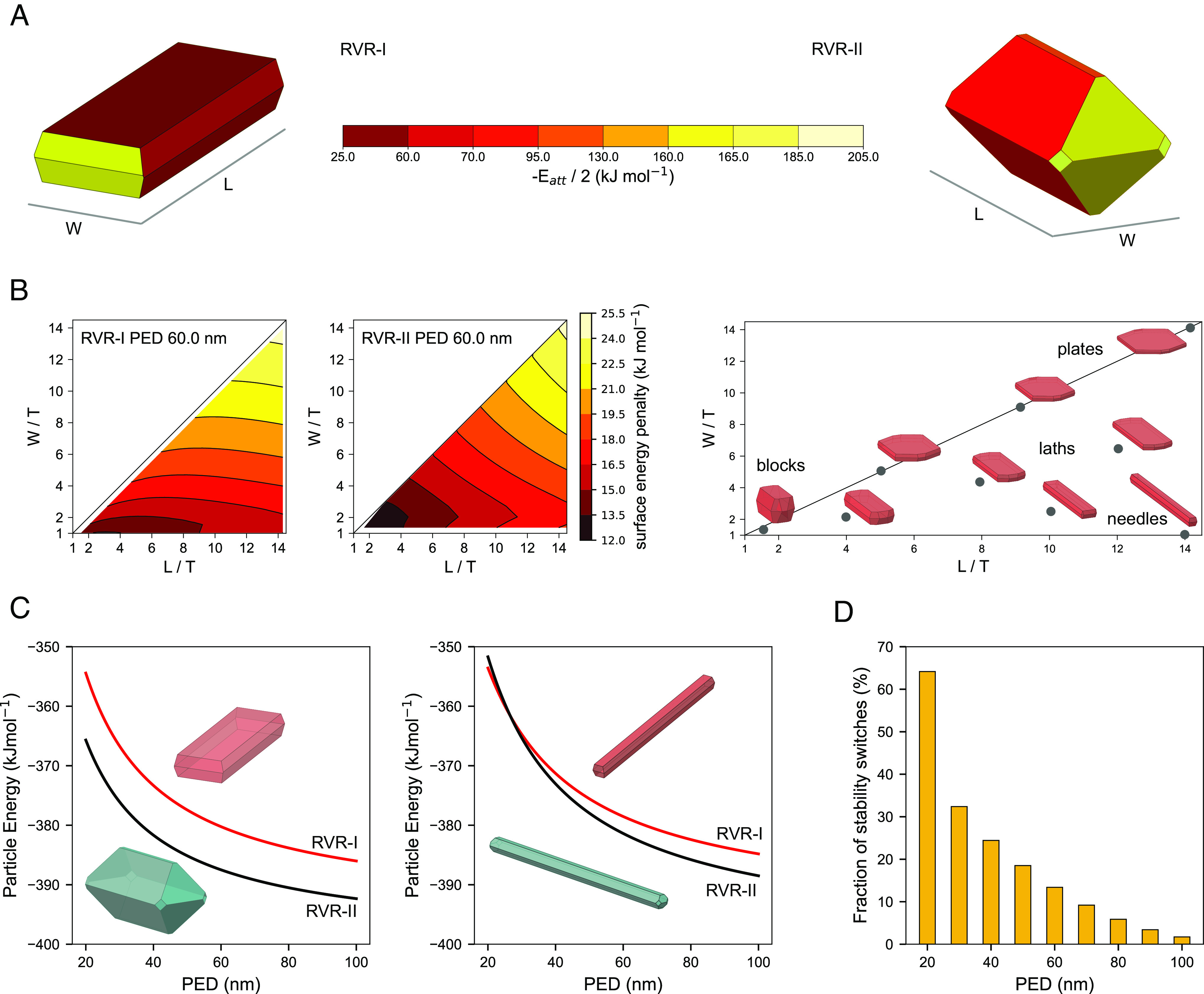
The thermodynamic stability of RVR polymorphs at the nanoscale depends on the relative destabilization due to crystal surfaces, which in turn is a function of the crystal size and shape. In general, RVR-II has higher surface energies. (*A*) Predicted attachment energy morphologies for RVR-I and II with facets colored according to their absolute surface energy penalty. (*B*) Surface energy penalties for RVR-I and II computed at a constant PED of 60 nm as a function of crystal shape (*Left*) and map of corresponding morphologies (*Right*). (*C*) Particle energy as a function of size for attachment energy morphologies (*Left*) and for arbitrary needle morphologies (*Right*). (*D*) Fraction of combinations of morphology pairs which afford a switch of thermodynamic stability between RVR-I and RVR-II (RVR-I becomes the stable form) at the given particle size.

Lattice energies for RVR-I and II were computed to be −395 and −400 kJ mol^−1^ respectively, with RVR-II computed to be the stable bulk polymorph. The surface energy penalty of RVR-I and RVR-II was then evaluated in order to compute the particle energy. Fig. 2*A* shows how the energy penalty of RVR-II surfaces is typically higher than the energy penalty of RVR-I surfaces. To illustrate the impact of the crystal shape on the crystal particle energy, the surface energy penalty for both RVR polymorphs was computed for 300 different crystal habits with L/T and W/T aspect ratios between 1 and 14 (where L, W and T are the length, width and thickness of the crystals) keeping the size of the particle constant at PED (particle equivalent diameter) 60 nm (Fig. 2*B*, where PED is the size of the diameter of an equivalent sphere with identical volume). We observed a strong dependence of the surface energy penalty with crystal shape. For both forms, the surface energy penalty is at its minimum for blocky crystals, and it increases as the particles become more elongated in either the L or W dimension. In Fig. 2*C*, the dependence of the particle energy as a function of size is then shown for both polymorphs adopting two sets of crystal morphologies. The particle energies of RVR-I and RVR-II do not cross when their morphologies are block-like (Fig. 2 *B*, *Left*) but they cross for needles (Fig. 2 *B*, *Right*). This shows that needles of RVR-I are more stable that needles of RVR-II at small sizes. Finally, we computed the probability of two random crystal morphologies within our defined aspect ratios to cross in energy as a function of PED size (>100 k computations). This probability is close to zero at sizes of 100 nm and increases up to 65% as the size decreases to 20 nm. The stability switch is most likely at small particle sizes (~40 nm) and for needle morphologies. We note that the latter represent the experimental morphologies typically observed for RVR-I and RVR-II both from solution crystallization (9, 28) and from our ball-milling experiments (Fig. 1*D*).

**Fig. 3.**
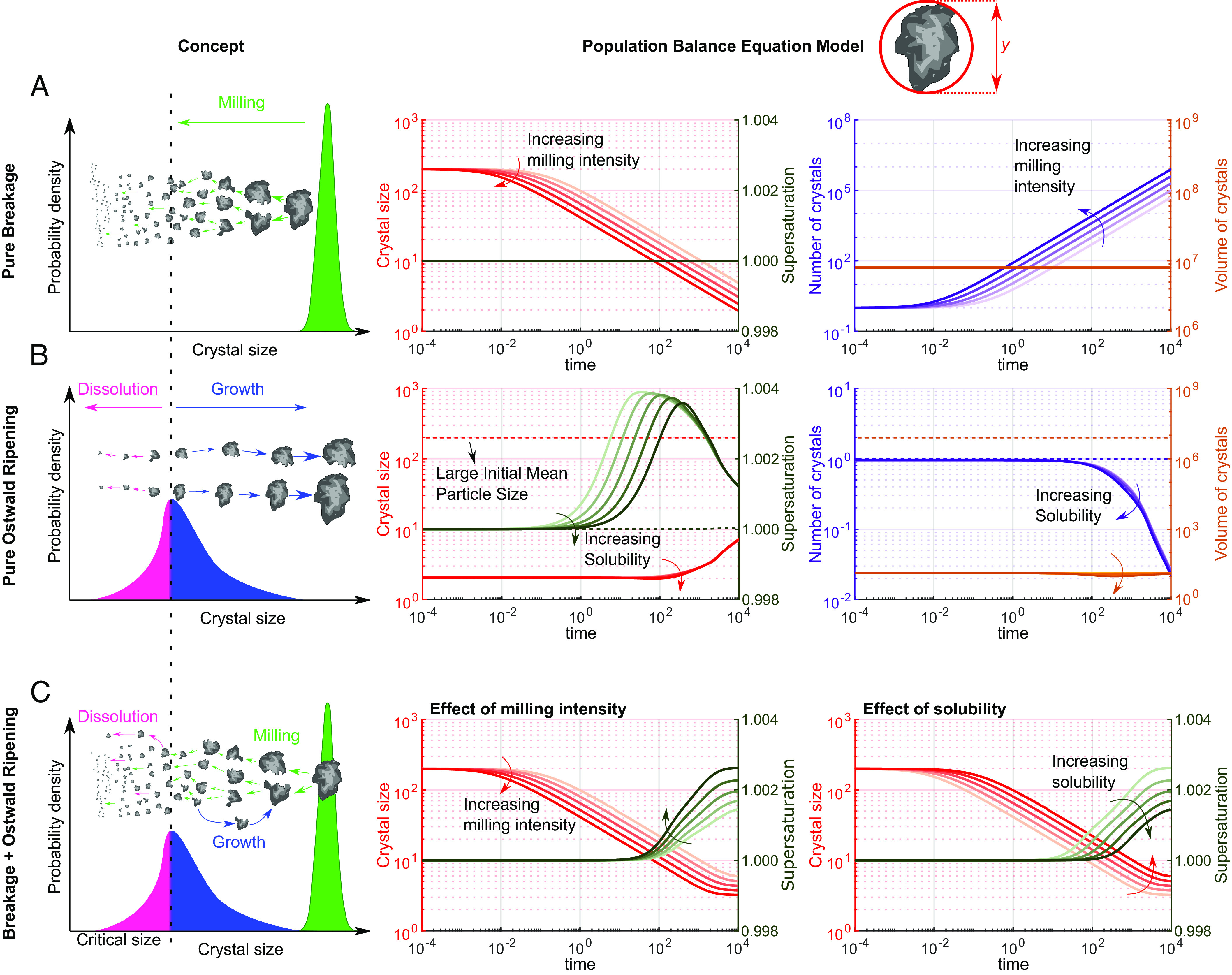
Qualitative evolution of the particle size distribution (column *Concept*) with the corresponding evolution of mean particle size, supersaturation, particle number, and particle volume for the three scenarios studied with PBEM. (*A*) Scenario with pure breakage. (*B*) Scenario with pure Oswald ripening. In this scenario, the dashed lines indicate the case of crystals with a large initial mean size. (*C*) Scenario with a combination of breakage and Ostwald ripening. The dashed vertical line in the *Left* column indicates the critical size below which dissolution occurs and above which growth occurs. In the plots, line colors are red for crystal size, brown for supersaturation, blue for number of crystals, and orange for volume of crystals.

To aid understanding the processes occurring in the mill, we modeled particle size evolution using population balance equation models (PBEMs) for three potential scenarios (Fig. 3 *A*–*C*) (29–31). The column *Concept* in Fig. 3 shows the qualitative evolution of the particle size distribution while the column PBEM shows the corresponding evolution of mean particle size, supersaturation, particle number, and particle volume for the three scenarios.

In the first scenario of pure breakage, milling results in a decrease in the mean particle size over time, and an increase in the number of particles. Increasing the milling intensity leads to a faster reduction in the particle size. In the second scenario of pure Ostwald ripening, crystals above the critical size eventually grow at the expense of the dissolution of crystals below the critical size (size below which crystals dissolve). As expected, in the absence of nucleation, the number of particles decreases over time due to dissolution, while the mean particle size and volume increase due to crystal growth. Overall, we note that in the absence of Ostwald ripening, we cannot expect generation of supersaturation (and in turn nucleation of a polymorph) and in the absence of particle breakage we cannot expect any growth/dissolution of crystals with large initial mean size (scenario b, dashed lines). Therefore, both these mechanisms were combined and the simulation outcome along with the concept is outlined in Fig. 3*C*. In the combined third scenario, the effects of milling intensity as well as solubility were studied. These simulations were performed under conditions such that, initially, particle breakage is the dominant mechanism, which is indeed a true reflection of our experiments (larger initial mean sizes). In the last scenario, we observe that the mean particle size and the supersaturation reach a steady-state value, due to the competing effects of simultaneous growth, dissolution, and breakage of crystals. This is in line with the experimental outcome, where particles reach a steady-state particle size with the variations of added solvent in the mill impacting the system’s solubility.

The above work sheds significant light on the mechanisms and thermodynamics of milling exemplified with the important system of RVR. The PBEM has shown that both breakage and Oswald ripening processes are required to explain the milling results. First, large crystals undergo significant size reduction in the ball mill through breakage until Ostwald ripening is triggered which leads to dissolution and growth of particles below and above a critical size, respectively. The steady-state particle size would be determined by the milling conditions including milling intensity as well as solvent type and concentration (which controls the solubility). Prolonged breakage together with Ostwald ripening establishes a steady-state particle size, with higher solubilities and lower milling intensities leading to larger sizes. The resulting steady-state size along with the supersaturation generated in the mill, will control the thermodynamics at the nanoscale, triggering nucleation and subsequently transformation to the more stable polymorph at the nanoscale.

With molecular modeling and milling, we have shown that not only size but also shape matters in this equilibrium. For RVR, the thermodynamic stability reverses at very small sizes (~40 nm), and for needle crystals, observed in the milled products with SEM. This observation reinforces the necessary role of Ostwald ripening and crystal growth in the mill (leading to needles) beyond the sole role of breakage (leading to blocks). In conditions of abundant solvent and high solubility (large steady-state size), RVR-II becomes the most stable phase and is produced readily from RVR-I since abundance of solvent accelerates nucleation and growth. In conditions of lower solvent volumes and low solubility (small size steady state), RVR-I becomes the most stable and can be produced from RVR-II, but it requires long milling times to achieve the steady state. In conditions of no solvent and very low solubility (very small particle size steady state), RVR-A becomes the sole phase. Conditions in between, lead to mixtures of phases. Our results are reproducible, demonstrating that mechanochemistry represents a reliable and consistent tool for polymorph discovery even for conformational phases.

Finally, molecular conformation plays a crucial role in switching thermodynamic stabilities of RVR polymorphs in the mill. As surfaces are generated in milling conditions affording smaller crystals, molecules are stripped of the stabilizing intermolecular interactions in the bulk and the conformer energy becomes more dominant. For this reason, at very small particle sizes, the two phases containing the stable trans conformer become thermodynamically favored (RVR-I and RVR-A). Remarkably, milling forces provide sufficient energy to convert between the trans and the cis conformers in the solid state despite the high energy barrier for the interconversion (>90 kJ mol^−1^).

The observation that RVR-I readily converts to RVR-II after milling in different solvents raises another important question, that is, whether it is possible that the initial formation of RVR-II during industrial manufacturing in Abbott’s laboratories was in fact due to a phase transition of RVR-I caused by mechanical action, which produced the first crystal seeds of RVR-II. Unfortunately, this hypothesis would be difficult to verify. It remains true, however, that RVR-I could have been regenerated from RVR-II by milling with the appropriate conditions, perhaps limiting, or avoiding the economic burden suffered by Abbot in the past.

To conclude, the results presented here have shown that breakage and Ostwald ripening processes dictate steady-state crystal size in the mill, that shape as well as size matter in the thermodynamics of nanocrystals and that polymorphs with high energy conformers become thermodynamically unfavored at the nanoscale. All these point toward a strong prevalence of polymorph stability switches at the nanoscale for conformational polymorphs and to ball-milling as a unique tool to explore polymorphism of complex flexible compounds, especially when guided by computer calculations of particle energies as a function of crystal size and shape. Mechanochemistry has been shown here to offer unprecedented levels of control on the crystal polymorphism in RVR, with its stable form easily nucleated and its metastable disappearing form easily recovered upon simple changes in environmental milling conditions.

## Materials and Methods

### Experimental Methods.

#### Materials.

RVR (98%) was purchased from Sigma Aldrich as pure RVR-I and used without further purification. RVR-II was produced pure by slow evaporation from ethyl acetate solutions. All solvents used were of analytical grade and used as received. Acetonitrile (MeCN, ≥99.9%) and 2-propanol (IPA, ≥99.5%) were purchased from Honeywell Research Chemicals, acetone (Ace, ≥99.8%) from Fisher Scientific UK Ltd, and ethyl acetate (EA, 99.8%) from Sigma Aldrich.

#### PXRD.

PXRD patterns of all samples were collected on a Bruker D2 Phaser diffractometer equipped with a LYNXEYE detector, using Cu-Kα radiation (λ = 1.54 Å). Intensity data were recorded in the 2θ range of 5 to 40°. Quantitative phase analysis and crystal size estimates were obtained by Rietveld refinement of collected data with TOPAS v5 (*SI Appendix*, section 2) (32, 33). Briefly, the peak shape and the parameters describing the diffractometer geometry were optimized using NIST 660b LaB6 standard and only a Lorentzian Scherrer term for each phase was modeled in the pseudo-Voigt functions for the quantitative analysis (the other parameters being fixed). A shifted Chebyshev function with six parameters was used to fit the background. To note is that the resulting Scherrer nanocrystal size from this method is independent of agglomeration since it provides the size of the nanocrystals (which leads to peak broadening effects) and not of agglomerated particles (which do not lead to peak broadening effects). The amorphous content of milled samples was determined using corundum (α-Al_2_O_3_) as an internal standard (34).

#### Solid-state NMR (SSNMR).

SSNMR experiments were performed using an Oxford 11.7 T superconducting magnet, and a Bruker AVANCE III HD console, operating at frequencies of 499.69 and 125.65 MHz for ^1^H and ^13^C, respectively. Approximately 100 mg of the sample was packed into a zirconia 4 mm rotor with a Kel-F cap. The CP-MAS with total sideband suppression (35) pulse sequence was used for spectral acquisition at a spin rate of 10 kHz using a 4 mm HX magic-angle spinning probe at 25 °C. The magic angle setting was calibrated using KBr. Spectra were obtained using a recycle delay of either 4 s (form I and amorphized form I) or 6 s (form II and amorphized form II) over 512 (form I and form II) or 800 (amorphized form I and amorphized form II) scans. The ^13^C chemical shifts were referenced using the high-frequency signal of adamantane [δ_iso_(^13^C) = 38.5 ppm]. SPINAL64 (36) heteronuclear decoupling was used during acquisition with a ^1^H nutation frequency of approximately 73.5 kHz. All spectra were processed in Topspin.

#### FTIR spectroscopy.

Solid-state FTIR spectra were recorded using a Thermo Fischer Scientific Nicolet iS50 FTIR Spectrometer.

#### SEM.

SEM images were collected with a Quanta 200 microscope operating under vacuum at 12.5 kV (spot size 2.5). Milled powder samples were dispersed on conductive tape on top of aluminum stubs and coated with 13 nm of platinum.

#### Ball-mill grinding experiments.

Ball-mill grinding experiments were performed with a Retsch MM400 Mixer Mill with screw top 5 mL stainless steel milling jars fitted with a Teflon (polytetrafluoroethylene, PTFE) gasket and 7 mm stainless steel milling balls using a milling frequency of 30 Hz. The duration, as well as the solvent used for each experiment varied as described below. The amount of solvent used is reported using the concentration h (solvent volume/mass of powder, µL mg^−1^). The ball milling protocols of Belenguer et al. (15) were followed throughout all experiments and phase composition was determined from PXRD data.

For the initial fast milling screening, NG and LAG experiments were performed with milling times of 45 min starting from 200 mg of either RVR-I or RVR-II. LAG experiments were conducted in presence of 50 µL (h = 0.25 µL mg^−1^) of various solvents. NG from both forms of RVR resulted in considerable amorphization of the samples with no polymorphic changes. LAG of the metastable polymorph RVR-I with IPA, MeCN, Ace, and EA resulted in the transformation to the bulk-stable RVR-II, whilst LAG with water afforded no polymorphic transformation and little to no amorphization. LAG of RVR-II with any of the mentioned solvents did not result in any polymorphic transformation except in the case of water, for which a transition to RVR-I was observed only if longer milling times (>45 min) were used.

We then conducted LAG experiments with IPA for prolonged milling times in order to establish whether a steady state for the observed polymorphic transformation of RVR-I to RVR-II had been reached. For different concentrations of IPA, a steady state was attained after just 15 min of milling, although the composition of the resulting products varied considerably depending on the concentration h (Fig. 1 *B*, *Upper*). At h = 0.05 µL mg^−1^ no conversion to RVR-II was observed; at h = 0.10 µL mg^−1^ the steady-state product contained a mixture of crystalline RVR-I and RVR-II which was later shown to also contain RVR-A; at h = 0.25 µL mg^−1^ a full conversion of RVR-I to RVR-II was attained. The time required for the LAG transformation of RVR-II to RVR-I in the presence of water (h = 0.25 µL mg^−1^) was also studied and was found to be much greater. The full conversion of RVR-II to RVR-I under these conditions typically required about 150 min of milling times.

The found milling conditions allowed us to repeatedly interconvert between RVR-I and RVR-II in full one-pot conversion cycles (i.e., LAG of RVR-I with IPA h = 0.25 µL mg^−1^ for 15 min to yield RVR-II; followed by LAG of RVR-II in water h = 0.25 µL mg^−1^ for 150 min to yield RVR-I).

The effect of different solvents and different compositions on the LAG equilibrium milling curves for the polymorphic conversion was also investigated. LAG to steady state should lead to identical milling outcomes independent of the RVR form used initially, only dependent on the milling conditions. Because of that, experiments were performed starting from RVR-I since the milling times to achieve steady state were established to be shorter (45 min). Equilibrium milling curves were derived by milling RVR-I with five different solvents (water, IPA, MeCN, Ace, EA) at five different solvent concentrations (from 0.05 to 0.25 µL mg^−1^) for 45 min (Fig. 1*C*).

#### Analysis of RVR-A.

The presence of RVR-A was verified by quantitative phase analysis of the milling product of RVR-I with IPA at 0.10 µL mg^−1^ for 45 min. These milling conditions were selected for analysis because LAG in IPA had the steepest milling equilibrium curves for the RVR-I to RVR-II transformation compared to other tested solvents (thus the least amount of RVR-A is to be expected). At these LAG conditions, the milling product was determined to be composed of 45% RVR-I, 30% RVR-II, and 25% RVR-A.

Further to this, pure RVR-A was produced by NG for a prolonged time (120 to 155 min) from RVR-I and RVR-II. A range of characterization techniques (PXRD, FTIR spectroscopy, and ^13^C SSNMR) were used to establish that RVR-A obtained from RVR-I and RVR-II were identical phases.

### Computational Methods.

#### Calculation of particle energies.

The thermodynamic stability of nanosized RVR crystals as a function of particle size (*r*) and shape (ϕ) was calculated using Eq. **1**.

The first term represents the stabilizing (negative) contribution of the bulk crystal structure, and the second term represents the destabilizing (positive) contribution of the crystal surfaces. *E_latt-inter_* is the intermolecular component of the lattice energy. *ΔE_intra_* is the intramolecular conformational energy penalty. *E_att_^(hkl)^* is the attachment energy of the (*hkl*) facet. The effect of particle size and shape on the particle energy is expressed through the fraction of surface molecules on each (*hkl*) facet, *x*_(*hkl*)_ (*r*, ϕ). Our model assumes crystal particles with idealized shapes and excludes the contribution of surface relaxation and edge energies to the surface energy penalty.

Particle energies were calculated using our Particle Energy Calculator (PEC) (37) code written in Python 3, which utilizes modules of the CSD Python API (38). Briefly, PEC takes as an input the crystal structure, the (*hkl*) attachment energies and an optional user-defined crystal morphology. The main task of PEC is to compute the fraction of surface molecules, *x*_(*hkl*)_ (*r*, ϕ), by calculating the volume of convex hulls that are scaled according to the input size. The program can run in two modes: i) generate several crystal morphologies with varying aspect ratios and calculate the particle energy for a user-defined particle size (volume) and ii) use a starting morphology to calculate the particle energy as a function of size in a range defined by the user. The results presented in this work were obtained using both modes.

A derivation of our model and details about the calculated energy terms and the working principles of the PEC code are presented in Supplementary Information.

#### DFT calculations.

Periodic DFT calculations of lattice energies (*E_latt-inter_*) and attachment energies (*E*_att_^(^*^hkl^*^)^) were performed with the Vienna ab initio Simulation Package (VASP 5.4.4) (39–41) with the Perdew–Burke–Ernzerhof (42) functional and Projector Augmented Wave (43, 44) potentials release 5.2, using a cut-off energy of 520 eV and applying the Tkatchenko–Scheffler dispersion correction method (45). The experimental crystal structures with CSD (38) refcodes YIGPIO02 (RVR-I) and YIGPIO03 (RVR-II) were used as starting models and their geometries were optimized with tight convergence settings (10^−8^ eV), relaxing both the unit cell parameters and the atomic positions. For the disordered crystal structure of RVR-I (YIGPIO02) the disorder component having the higher stability after optimization was considered. The intramolecular conformational energy terms of RVR-I and RVR-II, *ΔE_intra_*, were computed as described in Beran et al. (46).

#### PBEM.

The evolution of the state of the solid phase and of the liquid phase during milling were described using a PBEM. The model was formulated under the hypothesis that during milling the large crystals undergo significant size reduction through breakage. Upon size reduction, Ostwald ripening is triggered, which leads to dissolution and growth of particles below and above a critical nucleus size (*y*_n_), respectively. The objective of this model was to verify whether prolonged particle breakage and Ostwald ripening can establish a steady state in both particle size and solute concentration.

The PBEM developed in this work accounts for breakage and size-dependent growth and dissolution (Ostwald ripening) (29, 30). The breakage term in our model was simplified and crystal nucleation was assumed negligible. Crystals were assumed to be spherical and described by a single characteristic length (their diameter). Finally, the system was assumed to be composed of particles of a single solid phase. Although we observe polymorphic transformations in the RVR systems studied here, this assumption was made to highlight the interplay between growth, dissolution, and breakage mechanisms in reaching a final steady state of particle size and solute concentration. Note that due to the lack of experimental kinetic data, the PBEM was cast in a nondimensional form given by

[2] $$\frac{\partial n}{\partial \tau }+\frac{\partial (G^{*}n)}{\partial y}=B-D,$$

where the first term describes the evolution of the number density function *n* over the dimensionless time *τ*, the second term accounts for the growth/dissolution of crystal ensemble along their internal coordinate *y* with a nondimensional size-dependent velocity G* (growth/dissolution rate), and the terms on the right-hand side accounts for the birth *B* and death *D* of crystals upon breakage. The fundamental equations required to reproduce our model are reported in Supplementary Information, where both the dimensional and the nondimensional models are presented. The PBE was solved numerically using a fully discrete high-resolution finite volume method (47–49). Additional details on the solution methodology are provided in *SI Appendix*.

## Supplementary Material

Appendix 01 (PDF)

## Data Availability

The PEC code and Population Balance Modeling code are available on Github (https://github.com/quimiquilla/ritonavir_milling) (37). All data that support the findings of this study are available within the paper and its Supplementary Information, or from Durham University’s data repository (DOI: 10.15128/r26395w717f) (34).
