# Supplementary material for: Crystal size, shape, and conformational changes drive both the disappearance and reappearance of ritonavir polymorphs in the mill
Source: Proc Natl Acad Sci U S A. 2024 Apr 1;121(15):e2319127121. doi: 10.1073/pnas.2319127121 (PMC11009673; doi:10.1073/pnas.2319127121)
Supplement: Supplementary file 1 — Appendix 01 (PDF) [file pnas.2319127121.sapp.pdf]

PNAS

Supporting Information for

Crystal size, shape and conformational changes drive both the disappearance and reappearance of ritonavir polymorphs in the mill

Pietro Sacchi[a,b,1], Sarah E. Wright[a,1], Petros Neoptolemou[a], Giulio I. Lampronti[d], Ashwin Kumar Rajagopalan[a], Weronika Kras[a,c], Caitlin L. Evans[e], Paul Hodgkinson[e] and Aurora J. Cruz-Cabeza*[a,c,e]

[1] These authors contributed equally.

Author affiliations:

[a] Department of Chemical Engineering, University of Manchester, Manchester, UK, M13 9PL.

[b] The Cambridge Crystallographic Data Centre, Cambridge, UK, CB2 1EZ.

[c] Chemical Development, Pharmaceutical Technology & Development, AstraZeneca, Macclesfield, UK, SK10 2NA.

[d] Department of Earth Sciences, University of Cambridge, Cambridge, UK, CB2 3EQ

[e] Department of Chemistry, Durham University, Durham, UK, DH1 3LE

* **Corresponding author:** Aurora J. Cruz-Cabeza

Email: aurora.j.cruzcabeza@durham.ac.uk

This PDF file includes:

Supporting text
Figures S1 to S36
Tables S1 to S10
SI References

Supporting Information Text

Supporting Information Text 2
1. The Ritonavir (RVR) system 2
1.1. Conformational polymorphism of RVR 2
1.2. Crystal structure of RVR polymorphs 2
2. Experimental 3
2.1. Thermal analysis of RVR samples 3
2.2. Quantitative Phase Analysis of crystalline powders 3
2.3. Quantification of Amorphous Content 3
2.4. PXRD patterns used for constructing the thermodynamic equilibrium curves 4
2.5. PXRD patterns used for kinetic analysis 4
2.6. Characterisation of Amorphous Material 4
2.7. Amorphous phase under LAG ball mill conditions 5
2.8. Crystallite size of milling products 5
3. Molecular modelling 5
3.1. Intermolecular interaction energies of RVR polymorphs 5
3.2. Simulated IR spectra of optimised RVR conformers 6
3.3. Definition of particle energy 6
3.4. Calculation of lattice energies 7
3.5. Calculation of attachment energies 8
3.6. The Particle Energy Calculator (PEC) code 8
3.7. Influence of particle shape 8
3.8. Influence of solvent 9
3.9. Thermodynamic stability switch as a function of particle size 9
4. Population balance equation model 10
4.1. Breakage terms 10
4.2. Growth and dissolution terms 10
4.3. Scaled model 11
4.4. Conditions and parameters 12
4.5. Solution 12
Supplementary Figures and Tables 13

1. The Ritonavir (RVR) system

1.1. Conformational polymorphism of RVR

An analysis of the molecular conformations found in RVR-I and RVR-II is presented in Supplementary Figure 1. For simplicity, we only name the rotatable bonds which are significantly different between forms I and II. The central core rotatable bonds remain very similar between forms but the rotatable bonds determining the orientation of side groups either on the left, middle or right-hand side (L-side, M-Ph, R-side) of the molecule change significantly. The values of six relevant torsions are shown and classified as usual or unusual according to adjustment or change observations.[1] Amongst them, we focus our attention on the carbamate group (highlighted in yellow) which adopts a *trans* configuration in RVR-I and a *cis* configuration in RVR-II. The carbamate group configuration is central to the polymorphism of RVR. Trans is the stable configuration about the carbamate with an energy barrier for interconversion to the cis carbamate of nearly 90 kJ/mol (depending on the method of computation).[2]

1.2. Crystal structure of RVR polymorphs

RVR is a chiral compound and thus crystallises in two chiral space groups: RVR-I with a more stable trans conformer in $P2_1$ and RVR-II with a less stable cis conformer in $P2_12_12_1$. RVR forms

very strong hydrogen bonded chains (HBC) in both polymorphs (Supplementary Figure 2). In RVR-I, the molecules stack efficiently on top of each other forming three hydrogen bonds propagated via translation symmetry along the b-axis. In RVR-II, the molecules form four hydrogen bonds making use of roto translation (screw axis) symmetry along the a-axis.
The HBC interaction is significantly more stabilising than any of the other interactions in the crystal. Because of this, the HBC direction grows faster than the other crystal directions resulting in distinct needle morphologies for RVR-I and RVR-II from crystallisation. From crystallisation experiments in our lab, we have also observed RVR form gels when crystallised from certain solvents and under slow evaporation conditions. This is also consistent with a very strong and dominant interaction along one direction. The molecule-molecule interaction in the HBC was calculated to be -169.8 and -244.5 kJ/mol for forms I and II respectively, as calculated with PIXEL (see Section 3.1 below). The next most stabilising interaction in the crystal is -88.9 and -45.8 kJ/mol for forms I and II respectively, considerably less stabilising than the HBC interaction. The molecule-to-molecule distance in the HBC is 5.2-5.3 Å in form I (*b*-length) and 4.9-5.0 Å in form II (half of *a*-length) – considering the structures at 100 K (YIGPIO02 and YIGPIO03) and 300 K (YIGPIO and YIGPIO01).

2. Experimental

2.1. Thermal analysis of RVR samples

Differential Scanning Calorimetry (DSC) measurements on RVR samples were performed using a TA instruments DSC2500 with a heating rate of 5 °C/min. The normalised DCS traces for the two polymorphs are shown if Supplementary Figure 3, and the temperatures and enthalpy changes corresponding to the melting of the two polymorphs are reported in Table S1.

2.2. Quantitative Phase Analysis of crystalline powders

Quantitative phase analysis of crystalline powders was performed for samples containing polymorphic mixtures using TOPAS v5.[3,4] The simulated powder patterns of the crystal structures for the two polymorphs of Ritonavir were retrieved from the CSD[5] and then refined using a pure sample of each polymorph (Supplementary Figures 4 and 5). This step is useful for identifying the phases most affected by preferred orientation. For RVR-II these were (001) and (011), no preferred orientation was identified for RVR-I. The March-Dollase model for preferred orientation was applied on these crystal planes in the quantitative analysis. The amorphous fractions were assumed to be negligible for both systems. Furthermore, the peak shape and the parameters describing the diffractometer geometry were optimized using NIST 660b LaB_6 standard: only a Lorentzian Scherrer term (CS_L) for each phase was modelled in the Pseudo-Voigt functions for the quantitative analysis, the other parameters being fixed. A minimum limit of 30 nm for the crystallite size was defined to avoid correlations with the background. A shifted Chebyshev function with six parameters was used to fit the background. Rwp and $\chi 2$ values ranged typically from 9% to 13% and from 2 to 5 respectively.

2.3. Quantification of Amorphous Content

The amorphous content may be quantified by use of an internal standard, where a known quantity of a known phase is added to the crystalline powder. If an amorphous phase is present in the sample all the crystalline phases will be overestimated, and background is not considered. Real amorphous fraction in the sample can be calculated directly from:

$$W_i = [1 / (1 - W_s)] \cdot [1 - (W_s / W_{s,c})] \quad \text{(S1)}$$

where W_s is the experimental weight fraction of internal standard, while $W_{s,c}$ is the calculated weight fraction of internal standard from the refinement. The reliability limit for amorphous phase is generally considered 10%. The main problem for a quantification method with powder diffraction is choosing the internal standard, since it has to have the following features:

- it must have a microabsorption value as near to that of the sample as possible;
- it must have a good to optimum crystallinity;
- It must be inert in the analysed substance (also in grinding);
- It is better if its main diffraction peaks don't overlap on those of the sample;

- It is better if it doesn't present strong preferred orientation.

Corundum, α-Al_2O_3, was chosen as internal standard. Two mixtures of corundum with the product from form-I milled with 20ul of IPA for 45 minutes, with 20.2% (Sample A) and 36.3% (Sample B) wt of corundum.

2.4.PXRD patterns used for constructing the thermodynamic equilibrium curves

Supplementary Figures 8-12 show the normalised PXRD patterns used to contruct the LAG milling equilibrium curves shown in Figure 1b of the main text.

2.5.PXRD patterns used for kinetic analysis

Supplementary Figures 13-16 show the normalised PXRD patterns used to construct the LAG milling curves analysing the kinetics of the polymorphic transformation from RVR-I to RVR-II as shown in Figure 1c of the main text.

2.6.Characterisation of Amorphous Material

To ascertain whether the amorphous state obtained by NG starting from form I is equivalent in nature to the amorphous state obtained by NG from form II, we used longer milling times (120 min) to ensure no crystalline material remained. Milling for 120 min resulted in the pure amorphous state with no crystalline state remaining, starting from both forms I and II RVR. PXRD patterns and FTIR spectra of RVR forms I and II as well as the amorphous material obtained by NG from RVR-I and RVR-II are given in Supplementary Figures 17 and 18, respectively. The FTIR spectra indicate that both amorphous materials produced are very similar to each other and independent of the starting form. The FTIR of amorphous RVR also appears to be more similar to that of RVR-I than RVR-II, noting only a single broad peak at 1627 cm^{-1} and no peak at 1343 cm^{-1} suggesting a dominating presence of the *trans* conformer in the amorphous state. These FTIR spectra can also be compared to simulated IR spectra of the optimised gas-phase conformations (presented in Section 3.2 of this document), which show a striking similarity to their respective solid state FTIR spectra and suggests the position of these carboxyl stretches are indicative of the conformation within the structure.

Solid-state ^{13}C NMR was also performed for both NG products and compared to the crystalline RVR-I and RVR-II forms (Supplementary Figure 19). The NMR bands were found to be identical for both amorphous products as obtained from RVR-I and RVR-II, with some sharp peaks remaining for the products obtained from both crystalline forms. This suggests that some small crystalline clusters remain in the amorphous samples, more clearly present in the NG product from RVR-II. We note that samples had to be transported from Manchester to Durham for the NMR analysis and that in the days of transport a small fraction of the pure amorphous material had converted back to the form it was produced form. Crystalline RVR-I and RVR-II show clear differences in the NMR spectrum with the spectrum of RVR-I being a better match with the bands of RVR-A.

When the amorphous samples were held at 60°C for two hours and then analysed by PXRD (Supplementary Figure 21), the two amorphous forms returned to the crystalline form from which they were obtained from. Interestingly, the amorphous form obtained from form-I returned to form I much more rapidly than the amorphous form obtained from form II returned to form II (after the same time in the oven the NG-form II sample remains largely amorphous with only small peaks of form-II). Our interpretation of this is the following. The amorphous form contains a population rich in the *trans* conformer thus showing a higher spectroscopic similarity with form I. When the amorphous material is kept at high temperature for some time, there is enough energy for the molecules to return to the crystalline state. Small nanosized seeds of the previous forms (I and II) will serve as sites on which the crystalline material to reform. Thus, each amorphous material returns to its original form. Because the *trans* conformer being dominant in the amorphous state, the recovery of form I from the amorphous material is faster than the recovery of form II. For seeds of form II to grow, RVR needs to go through a conformational change in the amorphous state from trans to cis first which clearly seems to impact the kinetics of the conversion.

In summary, these characterisation experiments show that i) RVR-A phases obtained by NG from RVR-I and RVR-II are equivalent, ii) that samples are dominantly amorphous but some small crystalline clusters/nanocrystals may remain despite the prolonged milling which then can serve as

seeds, and iii) that RVR-A is more similar to RVR-I in its local structure, including its molecular conformation (trans).

2.7. Amorphous phase under LAG ball mill conditions

To investigate the possibility of a three-way equilibrium between RVR-I, RVR-II and RVR-A, LAG ball milling of the amorphous materials was performed. Each of the amorphous samples underwent LAG ball milling for 45 min at 30Hz with IPA and water at 0.25 µl/mg. In all cases the amorphous material returned to its starting crystal form, regardless of the solvent used for the milling (water or IPA). Interestingly, if a mixture of amorphous materials was used (50% obtained from NG of form I and 50% obtained from NG of form II), after 45 min of LAG with IPA at 0.25 µl/mg only RVR-I was produced. Finally, full conversion to RVR-II from both amorphous forms was achieved from the amorphous samples but required higher IPA solvent concentrations.

2.8. Crystallite size of milling products

The products of the milling RVR-I for 45 min with IPA at η of 0.05 µl/mg, 0.10 µl/mg and 0.25 µl/mg were analysed by scanning electron microscopy (SEM) in Supplementary Figure 22. The 0.05 µl/mg IPA milling product consists of RVR-A and RVR-II. The milling product at 0.10 µl/mg IPA is a mixture of all three phases: RVR-A, RVR-I and RVR-II at 25%:45%:30%; some regions of the SEM image show aggregated lumps of particles whilst other regions show nicely dispersed long needles. At 0.05 µl/mg IPA, where RVR-II is obtained exclusively, aggregated RVR-II particles can be clearly appreciated. The 0.05 µl/mg sample has considerably smaller particles (less than 0.5 µm in length) than the 0.10 and 0.25 µl/mg samples.
The crystallite size (Scherrer) was then investigated for the equilibrium as well as the kinetic ball milling experiments. For the equilibrium experiments we look at the RVR-I to the RVR-II conversions under LAG with different solvents and after 45 minutes of milling. Here, we choose the lowest solvent concentration affording form II (>85%) and the highest concentration affording form I (>85%) for the analyses of crystals sizes -since these are the critical sizes of the polymorphs around the change of stability. As we can depict from Supplementary Figure 23a, RVR-I crystallites are typically around 70 nm in size under these conditions whilst RVR-II crystallites are larger at around 80 nm in size. The critical size for the polymorph interconversion must thus be in between those two values at ~75 nm. Crystal sizes at milling equilibrium are affected by the solvent used as well as by solvent concentration, because interactions of the solvent molecules with the nanocrystal surfaces must change surface stabilities. This is evident in the case of EtOAc. It seems that in the presence of EtOAc RVR-II crystal surfaces are relatively stabilized by solvent interactions with respect to other solvents. This dependence of equilibrium crystal size on the solvent nature and concentration under LAG conditions has been reported by the authors for other molecular compounds.[6] For the kinetic experiment, we explore the evolution of crystal size for RVR-I (the starting form) and RVR-II (the polymorph being generated) as a function of time. Because of the large peak overlap between RVR-I and RVR-II, crystal size has been considered reliable when the phase concentration is larger than >20wt% only. Under the conditions of the experiment, 0.25 µl/mg IPA LAG, >85% RVR-I has already converted to RVR-II just after 5 minutes of milling. Crystal size data for RVR-I is only reported thus up to 7.5 min of milling. Here we see that as the milling time progresses, RVR-I crystals get smaller whilst RVR-II crystals grow remaining around the 80 nm equilibrium size or slightly above.

3. Molecular modelling

3.1. Intermolecular interaction energies of RVR polymorphs

Intermolecular interaction energies of RVR-I and RVR-II were calculated for the DFT optimised crystal structures using the PIXEL[7] method available in the MiCMoS[8] platform. The molecular charge densities of both polymorphs used for the PIXEL calculations were calculated using Gaussian16[9] at the MP2/6-31G** theory level, as suggested in the PIXEL user manual. Energy vectors generated with the ProcessPIXEL[10] code were used to visualise the magnitude and orientation of principal intermolecular interactions in the RVR polymorphs (Supplementary Figure S24). The calculated energies are reported in Tables S3 and S4.

3.2.Simulated IR spectra of optimised RVR conformers

Simulated IR spectra of the optimised conformers found in the RVR polymorphs are calculated using Gaussian16 at the M06/6-31+(d,p) level of theory and scaled to obtain the fundamental frequencies.[11] Particular attention is made to the peaks in the 1600-1800 cm^{-1} region, relating to the carbonyl stretches of the ureido (green), amide (yellow) and carbamate (grey) functional groups (Supplementary Figure 25).

In both simulated IR spectra, the ureido carbonyl stretches at 1654 cm^{-1}, and in the solid FTIR of forms I and II of RVR at 1620 and 1611 cm^{-1} respectively. The ureido group also exhibits *cis-trans* isomerism. In RVR form I, the ureido group exists as the *cis* isomer and the carbonyl forms a hydrogen bond chain with the ureido amino group (N-H..O d = 3.13 Å). In RVR form II, the ureido group exists as the *trans* isomer and the carbonyl forms a hydrogen bond with the hydroxyl group (O-H..O d = 2.69 Å). The shorter distance and thus stronger hydrogen bond in form II, could explain why the ureido stretch in the solid state FTIR of RVR form II is at a lower frequency than in form I.

The amide group in the simulated IR spectra of form I and form II stretches at 1671 and 1705 cm^{-1} respectively. The lower frequency in form I can be explained by the intramolecular hydrogen bond that forms in the optimised conformer, the hydroxyl and amide groups move closer together to form a hydrogen bond. However, the distance between these two groups in the crystal is much larger (3.98 Å), and less likely to be the preferential hydrogen bond in the crystal, with a much shorter intermolecular interaction between the hydroxyl group and the Nitrogen of the thiazoyl group (2.99 Å) more likely. The solid FTIR of forms I and II gives 1645 and 1661 cm^{-1} respectively. In form I, the amide forms hydrogen bond chains (N-H…O d = 3.03Å) and in Form II the amide oxygen forms a hydrogen bond with the carbamate amino (N-H…O d = 2.88Å).

Finally, carbamate carbonyl stretches have been previously been reported to be in the region of 1690-1736 cm^{-1}, specifically with secondary carbamates stretching at 1705-1722 cm^{-1}.[12] This is lower than observed in our solid FTIR spectra of 1739 and 1757 cm^{-1} for forms I and II respectfully. In the crystal, form I forms carbamate hydrogen bond chains (N-H…O d = 3.13Å) and form II forms a hydrogen bond with the ureido amino group (N-H…O d=2.93Å). The simulated IR spectra reports lower frequencies of 1715 and 1703 cm^{-1} for form I and II respectfully, with no intramolecular hydrogen bonding involving the carbamate group being observed in either conformer.

3.3.Definition of particle energy

The particle energy depends on both of particle size (r) and shape (φ) and was defined as the sum of a stabilising energy term related to the formation of a bulk crystalline phase and a destabilising term due to the formation of surfaces:

$$E_{particle}(r,\phi) = E_{latt} + E_{Penalty}^{Surface} \tag{S2}$$

The lattice energy is the energy difference between a molecule in the crystal (with Z molecules in the unit cell) and the most stable gas phase conformer:

$$E_{latt} = \frac{E_{cry}}{Z} - E_{mol,gas} = E_{mol,cry} - E_{mol,gas} = (E_{latt-inter} + \Delta E_{intra}) \tag{S3}$$

The lattice energy can be considered as the contribution of an intermolecular term, Elatt-inter, and an intramolecular term, ΔEintra, which is the difference between the energies of an isolated molecule with the in-crystal conformation (E'mol,gas in Supplementary Figure 26) and of the optimised conformer (Emol,gas).

In equation S2, $E_{Penalty}^{Surface}$ represents the total destabilisation of the particle and is the weighted average of the contributions from all (*hkl*) crystal facets:

$$E_{Penalty}^{Surface} = w \sum_{(hkl)} E_{surface}^{(hkl)} \tag{S4}$$

Here, $E_{surface}^{(hkl)}$ is the energy felt by a molecule in the layer of a surface with Miller indices *hkl*. One way to define this energy is to consider it as the average of two contributions: one from the interaction of the molecule with the bulk (E_{latt}) and one from the interaction of the molecule with the crystal *hkl* slab it belongs to ($E_{slice}^{(hkl)}$):

$$E_{surface}^{(hkl)} = \frac{\left(E_{latt} + E_{slice}^{(hkl)}\right)}{2} \tag{S5}$$

Where the slice energy $E_{slice}^{(hkl)}$ is the energy difference between a molecule in a slab (with N molecules) of thickness d_{hkl} and an isolated molecule in the gas phase:

$$E_{slice}^{(hkl)} = \frac{E_{slab}}{N} - E_{mol,gas} = E_{mol,slab} - E_{mol,gas} = \left(E_{slice-inter}^{(hkl)} + \Delta E_{intra}\right) \quad \text{(S6)}$$

The slice energy is used to calculate the attachment energy[13] as:

$$E_{att}^{(hkl)} = E_{att-inter}^{(hkl)} = E_{latt} - E_{slice}^{(hkl)} = E_{latt-inter} - E_{slice-inter}^{(hkl)} \quad \text{(S7)}$$

We note that the attachment energy is just an intermolecular term. Thus, substituting equation S7 into equation S5, we get the following expression for the surface energy penalty of a *hkl* crystal facet:

$$E_{surface}^{(hkl)} = \left(E_{latt-inter} + \Delta E_{intra} - 0.5\, E_{att}^{(hkl)}\right) \quad \text{(S8)}$$

The particle energy can then be computed as:

$$E_{particle} = \left(\frac{N_{bulk}\left(E_{latt-inter} + \Delta E_{intra}\right) + \sum_{(hkl)} N_{surf}^{(hkl)}\left(E_{latt-inter} + \Delta E_{intra} - 0.5\, E_{att}^{(hkl)}\right)}{N_{total}}\right) \quad \text{(S9)}$$

Where N_{bulk}, N_{surf} and N_{part} are the number of molecules in the bulk and in the surface and the total number of molecules in the particle, respectively, and depend on the particle's size and shape. Finally, equation S9 can be simplified to equation S10, which was used in our model and which depends exclusively on the fraction of surface molecules on each facet, $x_{(hkl)}$.

$$E_{particle}(r,\phi) = \left(E_{latt-inter} + \Delta E_{intra}\right) - 0.5 \sum_{(hkl)} x_{(hkl)}(r,\phi) E_{att}^{(hkl)} \quad \text{(S10)}$$

3.4. Calculation of lattice energies

Periodic DFT calculations were performed using VASP 5.4.4 as described in the main text. Both the the Grimme GD2 dispersion correction method[14] and the Tkatchenko-Scheffler (TS) dispersion correction method[15] were tested. In both cases, the experimental crystal structures with CSD refcodes YIGPIO02 (RVR-I) and YIGPIO03 (RVR-II) were used as starting models and their geometries were optimised relaxing both the unit cell parameters and the atomic positions. For RVR-I, only the most stable component of the disorder was considered after the first optimisation step. Table S6 shows a comparison of the lattice parameters of the RVR polymorphs before and after DFT optimisation using the GD2 or TS correction methods. In both cases, the comparison was performed using the rmsd parameters described by Sacchi et al.[16]:

- Atomic positions of overlaid clusters of 20 molecules (rmsd-20)
- Unit cell lengths (rmsd-CL)
- Unit cell angles (rmsd-CA)

Overall, the optimised crystal structures of both RVR-I and RVR-II obtained with the TS model are more similar to the experimental crystal structures than the ones resulting from the GD2 calculations.

The intermolecular term of the lattice energy, $E_{latt-inter}$, was calculated for each polymorph as the difference between the electronic energy of a molecule in the crystal and the energy of an isolated gas-phase molecule having the in-crystal conformation (positioned in a 30 x 30 x 30 Å supercell) . The intramolecular energy term, ΔE_{intra}, was calculated as the difference between the energy of the most stable optimised gas-phase conformer (the *trans* conformer) and the gas-phase molecule having the in-crystal conformation. The ΔE_{intra} term was calculated also with the MP2D method of Beran et al.[17] Table S7 shows the calculated energies of the bulk RVR-I and RVR-II polymorphs.

The calculated lattice energies were compared to experimental values for the stability difference between RVR-I and RVR-II available in the literature. Bauer and Chemburkar reported the solubilities of RVR-I and RVR-II in several solvent mixtures,[18] which we used to calculate energy differences between the two polymorphs ($\Delta G = RT\ln(x_I/x_{II})$). The calculated energy difference from solubilities measured in ethanol/water mixtures was calculated to be 3.6 $kJmol^{-1}$ on average, while the energy difference from ethyl acetate/heptane mixtures was calculated to be 1.5 $kJmol^{-1}$ on average. In addition, the energy difference calculated from the enthalpy of melting of the two polymorphs (Table S1) was 3.3 $kJmol^{-1}$. Overall, the calculation using the TS dispersion correction paired with the MP2D correction yields the best approximation of the experimental values.

3.5.Calculation of attachment energies

For each polymorph of RVR, attachment energies of relevant *hkl* planes were calculated as the difference between the energies of a molecule in the crystal and of a molecule in a *hkl* slab of thickness d_{hkl} separated by vacuum slabs of at least 40 Å. The relevant hkl facets were identified using the BFDH method.[19] Implicit solvation effects on the attachment energies were calculated using the VASPsol module[20,21] with dielectric constants of 19.3 and 78.4 for isopropanol and water, respectively. The effect of implicit solvent models on the calculated attachment energies was small, with little impact on the calculated particle energies (see Section 3.8 of this document).

The calculated attachment energies and the total fraction of the morphology area (i.e., the morphological importance) for the *hkl* facets of RVR-I and RVR-II are reported in Table S8, while the resulting *vacuum* attachment energy morphologies are shown in Supplementary Figure . Inclusion of the implicit solvent has quite a profound effect on the calculated attachment energies, although the relative crystal morphologies show little change.

3.6.The Particle Energy Calculator (PEC) code

The Particle Energy Calculator (PEC) algorithm is written in Python3 and makes use of the CSD Python API.[5] It reads a crystal structure (from a CSD refcode or a CIF file), as well as the lattice and attachment energies calculated previously by the user. The main task of the PEC algorithm is the calculation of the fraction of surface molecules $x_{(hkl)}(r,\phi)$ for each of the relevant crystal surfaces. First, an assumption of the crystal habit (ϕ) is required. This can be any morphology specified by the user. Alternatively, if no user input is provided, the growth morphology calculated from the attachment energies is used.

The input morphology will determine the relative facet distances which are scaled according to the desired input particle size (or series of sizes) and used, together with the facet plane normal vectors, as input for the Particle Properties Calculator (PPC) module. This module, also developed in house, computes the intersections between the planes of the particle's facets and returns the volume and surface of the morphology convex hull. The fraction of surface molecules for each facet, $x_{(hkl)}(r,\phi)$, is then calculated as:

$$x_{(hkl)}(r,\phi) = \frac{w_{(hkl)}(V_{particle} - V_{core})}{V_{particle}} \tag{S11}$$

Where $w_{(hkl)}$ is the facet's morphological importance (calculated for the input morphology) and, $V_{particle}$ and V_{core} are the volumes of the particle and the inner core of the particle respectively. The V_{core} is calculated by generating an "inner" particle by subtracting $d_{(hkl)}$ distances from every morphological (hkl) face and generating the resulting inner convex hull (Supplementary Figure 27). We note that this technique for the calculation of $x_{(hkl)}(r,\phi)$ fails for calculations performed on too small particles (roughly, < 20 nm) All considered, this doesn't represent an issue, as for particles smaller than this size, where the effect of crystal edges on the surface energy penalty cannot be disregarded,[22] the assumptions made for our model would not hold.

The particle size is then expressed as the equivalent diameter of a sphere having volume equal to $V_{particle}$. A schematic view of the PEC algorithm is shown in Supplementary Figure 28.

3.7.Influence of particle shape

Because the particle energy is a function of the fraction of molecules at the surface, $x_{(hkl)}(r,\phi)$, the relative surface energy penalty depends on the crystal shape as well as the crystal size. The PEC algorithm scales the relative facet distances of an input morphology so that the desired size is achieved. The input morphology is however fixed during the calculation, and the crystal habits that are chosen as starting points can have a strong influence on the relative particle energies. We have evaluated the effect of the initial morphology by calculating the surface energy penalties of particles having fixed volume but variable aspect ratios.

The L/T and W/T aspect ratios were used to describe the shape of each particle, where L, W and T are the length, width and thickness dimensions of the orthogonal bounding box surrounding the morphology as shown in Supplementary Figure 29. For RVR-I, the thickness T was defined as the smallest dimension of the bounding box of its growth morphology. In the case of RVR-II, instead, the thickness T was defined as being delimited by the {002} facets. An alternative definition of the

W and T dimensions to describe the shape of RVR-II by using the {011} facets is also possible (see Supplementary Figure 29c, T definition 1). In the PEC, the particle shape is defined solely by the facet distances specified by the user. If definition 1 is used for the T dimension, halving of the {011} distances results in particles with little change in the W/T ratio. On the contrary, choosing the {002} distances ((see Supplementary Figure 29c, T definition 2) to control the T dimension allows more flexibility for the definition of the RVR-II particles' shape and a larger variety of different aspect ratios can be explored.

The effect of different particle shapes was evaluated by generating particle morphologies with L/T and W/T aspect ratios between 1 and 14.5, with L/T > W/T. In total, we have used 351 different particle morphologies for RVR-I and 290 for RVR-II. Supplementary Figure 30 clearly shows the calculated surface energy penalty for fixed particle volumes as a function of the particles' aspect ratios. For both polymorphs, the surface energy penalty is minimum for particles having a blocky morphology, and it increases as the particles become more elongated in either L or W direction. In the case of RVR-I, the energy penalty has a steeper dependence on the change of the W/T ratio compared to the L/T, whereas for RVR-II changing either of the two ratios has a similar effect.

Depending on the crystal morphologies selected for the calculation of particle energies, several scenarios are possible. Supplementary Figure 31 shows the particle energy as a function of size for three different cases. In case A the morphologies of both RVR polymorphs correspond to the morphology having the minimum surface energy penalty as shown in Supplementary Figure 30. In case B, both polymorphs have the respective attachment energy morphologies, i.e., the morphologies that are used by PEC if the crystal shapes are not defined by the user. Finally, case C refers to arbitrary selected needle morphologies.

3.8. Influence of solvent

The influence of solvent on particle energies was estimated with the use of implicit solvent models for the calculation of DFT-d attachment energies. The attachment energies of both polymorphs decrease as the solvent's dielectric constant increases (going from vacuum to IPA to water), and the particle energies decrease accordingly (Supplementary Figure 31). The effect of solvent is hardly noticeable when computing the particle energies of the two polymorphs, although RVR-I seems to be slightly favoured thermodynamically (see next Section).

3.9. Thermodynamic stability switch as a function of particle size

We have shown that a switch in thermodynamic stability for RVR polymorphs nanocrystals is indeed possible, with a careful choice of the initial particle morphologies and using accurate energies for both their lattice and the attachment energies. To provide more general conclusions, we have considered the requirement for the validity of our hypothesis, i.e., that a thermodynamic switch between the RVR polymorphs is possible at small particle sizes. To achieve such crossing the difference in particle energy between the RVR-I and RVR-II polymorphs, $\Delta E_{Particle}$, needs to be less or equal than zero. Because the lattice energy difference is constant and always positive, it follows that the condition for a switch in polymorph stabilities will be satisfied only when the negative difference between the surface energy penalties of the two polymorphs is greater or equal to the lattice energy difference (i.e., $-\Delta E_{Penalty}^{Surface} \geq \Delta E_{Latt}$). In other words, the destabilisation due to the surface of the particle of RVR-II compared to that of the particle of RVR-I needs to be larger than the lattice energy difference. This condition was tested for about 100 000 combinations of possible particle aspect ratios at different fixed particle volumes. The results are shown in Supplementary Figure 33, where the particle's volumes are expressed through the respective equivalent diameter, PED. When the particles are small, most of the combinations of aspect ratios will allow the particle energies to cross with the fraction of combinations that satisfy the crossing condition decreasing with increasing particle size. The effect of different solvents, included with an implicit solvent model used for the calculation of attachment energies, was also considered. In general, a higher dielectric constant (water > IPA > vacuum) seems to promote the thermodynamic switch at smaller particle sizes, while the effect at sizes > 50 nm is the same.

Supplementary Figures 34-36 show histograms of the aspect ratios of the RVR-I and RVR-II pairs of morphologies for which the stability switch condition is satisfied. These figures clearly show how the switch is predominant for needle particles (high L/T and low W/T) for both RVR-I and RVR-II at

small sizes, shifting to platelike morphologies at higher sizes, where a stronger surface energy penalty is needed to achieve a switch as the fraction of surface molecules decreases.

4. Population balance equation model

Below we present the population balance equations required to reproduce the model. First, we present the real model i.e., the model with units. Then, we show how the model is scaled to become dimensionless.[23,24] Finally, we briefly explain the solution of the model.

The model assumes that the crystals are spherical particles and can be described by one characteristic length, their diameter x. In addition, we assume that nucleation is negligible due to the extremely low supersaturation created in the mill. That supersaturation is created by dissolving particles with diameters smaller than the nucleus critical size. While particles larger than the nucleus critical size grow. In summary, our spherical particles undergo breakage, dissolution, and growth, defining the number density balance:

$$\frac{\partial f}{\partial t} + \frac{\partial (Gf)}{\partial x} = B - D \tag{S12}$$

where the first term $\frac{\partial f}{\partial t}$ is the number density change *f* over real time *t*. The second term $\frac{\partial (Gf)}{\partial x}$ is used to simulate the growth of particles i.e., the movement of *f* through dimension *x* with size-dependent velocity *G* i.e., growth rate. Birth *B* and death *D* terms are used to simulate the effect of breakage, where broken mother particles disappear, and new daughter particles appear in their place. The parameters used in our model are summarised in Section 4.4 of this document.

4.1. Breakage terms

The general birth and death terms are given by:

$$B = \int_x^\infty b(\eta) f(\eta, t) g(x, \eta)\, d\eta \tag{S13}$$

$$D = b(x) f(x, t) \tag{S14}$$

where a mother particle η is selected to die with breakage frequency *b(η)*. Then, two daughter particles are born whom their size is decided by the daughter distribution *g(x, η)*.

The breakage frequency is given by:

$$b(x) = p_m\, \zeta(x) \tag{S15}$$

where p_m is the parameter used to control the milling intensity (see Section 4.4 in this document), while $\zeta(x)$ is defined as:

$$\zeta(x) = x \left(1 + exp\left(-\frac{x - k_b}{x_{ref}}\right)\right)^{-1} \tag{S16}$$

which simulates two behaviours. First, size-dependent breakage where larger particles have a higher chance to break than smaller particles. Second, it provides a minimum size limit $k_b = 1$ at which breakage no longer occurs. The $x_{ref} = 1 \times 10^{-6}\, m$, is simply used for numerical stability.

The daughter distribution was simply given by:

$$g(x) = \frac{2}{x} \tag{S17}$$

where at one breakage event, two daughter particles form and have the same probability to have any size smaller than the mother particle. In addition, the equation makes sure that the summed volume of the daughter particles is equal to the mother's volume.

4.2. Growth and dissolution terms

The PBE equation is coupled with a mass balance equation:

$$\frac{dc}{dt} = -k_v \rho \frac{d\varphi_3}{dt} \tag{S18}$$

where the LHS represents the change of concentration *c* in the liquid phase which is equal to the RHS i.e., the change in mass in the solid phase. To convert the change in volume $\frac{d\varphi_3}{dt}$ to change in mass we multiply by the density ρ and shape factor k_v. For spherical particles $k_v = \frac{\pi}{6}$. The moments of the distribution are defined as:

$$\varphi_j = \int_0^\infty x^j f(x,t)\,dx \tag{S19}$$

Further, the size-dependent growth rate is defined. This is done by assuming that the supersaturation $S^*(x)$ is dependent on the particle size *x*. This way, we simulate the fact that the stability of the crystal is dependent on its size.

$$S^*(x) = \frac{c^*(x)}{c_\infty} = \exp\left(\frac{a}{x}\right) \approx \left(1 + \frac{a}{x}\right) \tag{S20}$$

where $c^*(x)$ is the solubility of the particle with size *x*, c_∞ is the solubility of the bulk liquid and the capillary length *a* is a term that combines all the physical parameters of the particle (see references 23 and 24 for more information). The equation is further simplified to $\left(1 + \frac{a}{x}\right)$, which comes from the first two terms of the Taylor expansion.

The growth rate equation is defined as:

$$G(x) = \frac{c_\infty^\lambda k x^\beta}{\rho}\left(S - S^*(x)\right)^\lambda \tag{S21}$$

where the parameters $\lambda = 1$ and $\beta = 0$ were chosen. The parameter could be set to $\beta = 1$ for a diffusion-limited mechanism, however our purpose to show the applicability of this model to ball milling is not affected, hence we chose $\beta = 0$ for simplicity. The supersaturation *S* is defined as the ratio of the bulk concentration *c* to bulk solubility:

$$S = \frac{c}{c_\infty} \tag{S22}$$

The initial conditions are defined as:

$$f(x,0) = f_0(x) \tag{S23}$$

$$c(0) = c_0 \tag{S24}$$

4.3. Scaled model

As mentioned in the main manuscript, due to the lack of experimental parameters we have scaled our model, which still matches our purpose to evaluate if the proposed mechanism can simulate the particle size and supersaturation evolution in the ball mill.

We used the following relationships to scale our variables:

$$y = \frac{x}{x_0}, \quad \tau = \frac{t}{t_0}, \quad a^* = \frac{a}{x_0}$$

Where x_0 is an arbitrary reference dimension and t_0 is the reference time, which is defined as:

$$t_0 = \frac{\rho x_0^{1-\beta}}{k c_\infty^\lambda} \tag{S25}$$

where $k = 0.5$ is the growth rate constant, chosen arbitrarily, $\beta = 0$ and $\lambda = 1$.

The daughter distribution and breakage frequency are calculated using the dimensionless size *y*, hence the breakage frequency becomes:

$$b^*(y) = b(x) x_0 t_0 \tag{S26}$$

The scaled supersaturation term becomes:

$$S^*(y) = \frac{c^*(yx_0)}{c_\infty} = exp\left(\frac{a}{x_0 y}\right) = \exp\left(\frac{a^*}{y}\right) \approx \left(1 + \frac{a^*}{y}\right) \tag{S27}$$

where the scaled capillary length was chosen arbitrarily[23] as $a^* = 0.01$.

The scaled growth rate then becomes:

$$G^*(y) = S - \left(1 + \frac{a^*}{y}\right) \tag{S28}$$

The liquid phase is now calculated by the change in supersaturation *S* which is one of the variables that were explored in our analysis:

$$\frac{dS}{d\tau} = -\frac{k_v \rho \chi_0^3}{c_\infty}\frac{d\varphi_3}{d\tau} \tag{S29}$$

The scaled PBE becomes:

$$\frac{\partial n}{\partial \tau} + \frac{\partial (G^* n)}{\partial y} = B - D \tag{S30}$$

The initial supersaturation conditions is given as:

$$S(\tau = 0) = S_0 = \frac{c_0}{c_\infty} \tag{S31}$$

From the relation $n\,dy = f\,dx$ we get $n_0 = x_0 f$, hence the initial condition of the scaled number density distribution becomes:

$$n(y, \tau = 0) = n_0(y) = x_0 f_0(y x_0) \tag{S32}$$

Finally, the solubility is controlled by the hold-up ratio *v*:

$$\nu = \frac{k_v \rho \mu_3}{c_\infty} \tag{S33}$$

To specify the initial volume of particles μ_3, the initial number of particles was fixed to $\mu_0 = 1$. The hold-up ratio was defined arbitrarily, and the solubility was calculated from the above equation.

4.4. Conditions and parameters

The conditions required to run the experiments are displayed in Table S9**.** All the initial distributions were assumed to be normal, described by their initial mean $\bar{y}_0$ and standard deviation σ_0. The arbitrary parameters used are reported in Table S10.

4.5. Solution

The PBE was solved using the Finite Volume Method using Strang splitting,[25] where the growth and dissolution fluxes were calculated according to Qamar et al.,[26] and the breakage fluxes were calculated according to Kumar and Kumar.[27] The bins of the grid for the particle size dimension Δy were set at 0.4, while tests at Δy = 0.1 showed no significant increase in accuracy. The Courant–Friedrichs–Lewy (CFL) condition was used to calculate the timestep Δt in each iteration.

Supplementary Figures and Tables

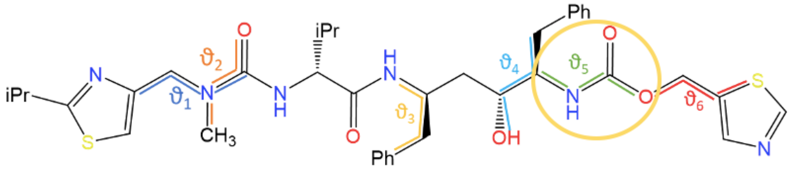

	Torsion #	Torsion ID	Δϑ (°)	Form I (RVR-I, YIGPIO02) Value (°)	Classification	Form II (RVR-II, YIGPIO03) Value (°)	Classification
**L-side**	ϑ_1	CC–NC	170.8	-64.2	Usual	125.0	Unusual Adjustment
	ϑ_2	OC–NC	171.2	156.8	Usual	-15.0	Usual
M-Ph	ϑ_3	CC–CN	111.6	178.3	Unusual Change	-70.1	Usual
**R-side**	ϑ_4	OC–CC	136.6	67.7	Usual	-68.9	Usual
	ϑ_5	OC–NC	175.5	178.7*	Usual	-5.8**	Unusual Change
	ϑ_6	OC–CS	107.3	52.8	Usual	160.1	Unusual Change

*Trans carbamate; **Cis carbamate;

Fig. S1. (Upper) Molecular structure of Ritonavir (RVR) with definition of torsion angles defined about rotatable bonds that change between forms I and II. (Lower) Table containing detailed information on torsion angle definitions, values and statistical conformational classifications for the two crystal conformations found in the two polymorphs of RVR. The torsion defined about the carbamate central bond is highlighted as yellow since the most significant conformational change in RVR occurs about this bond.

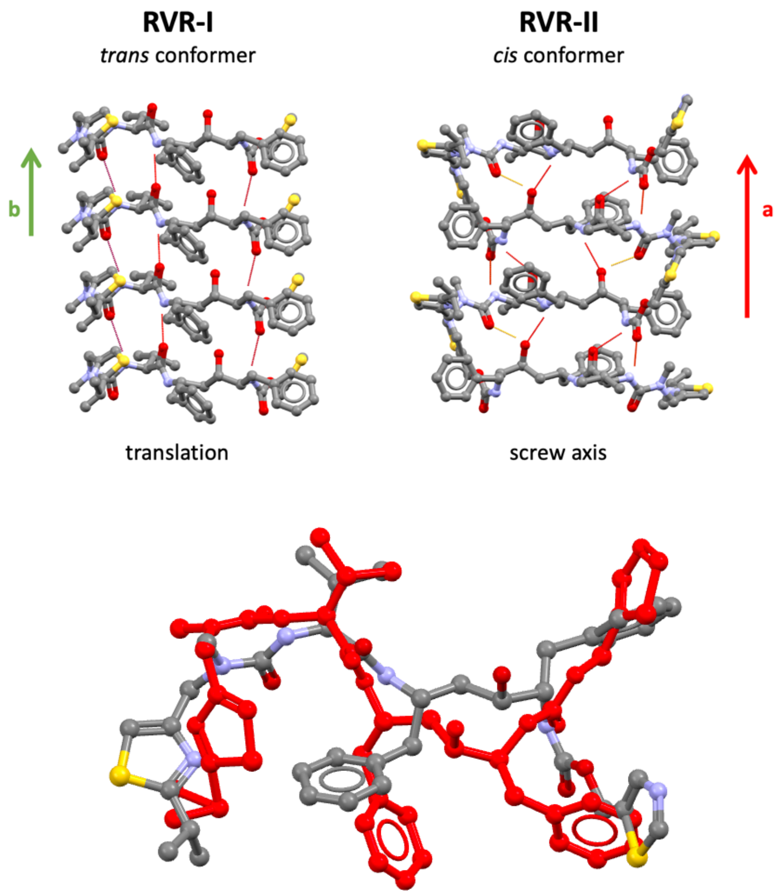

Fig. S2. (Upper) Packing view of the strong hydrogen bonded chains of RVR along the b-axis in RVR-I (YIGPIO02) and along the a-axis in RVR-II (YIGPIO03). (Lower) Overlay of the molecular conformations in RVR-I (YIGPIO02, trans-conformer, red) and RVR-II (YIGPIO03, cis-conformer, atom colouring).

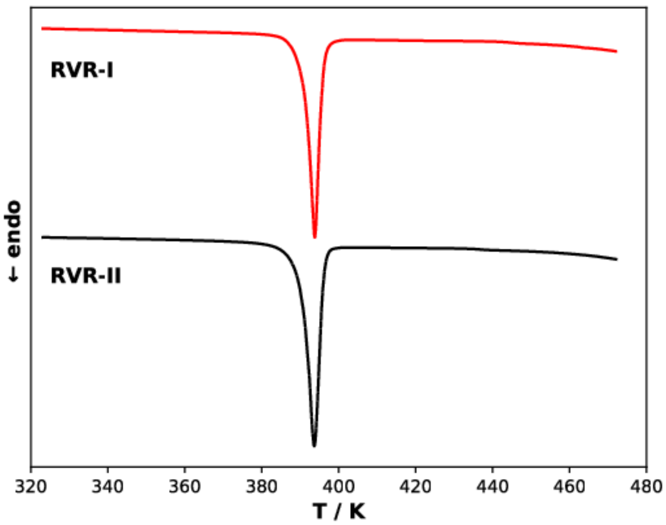

Fig. S3. Normalised DSC traces of RVR polymorphs

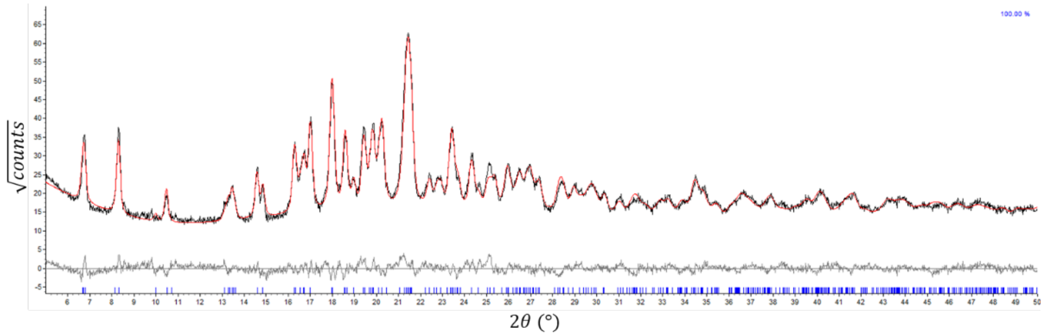

Fig. S4. Rietveld refinement of Ritonavir form I. Experimental (black curve), calculated (red curve) and difference (grey curve) patterns of Ritonavir form I.

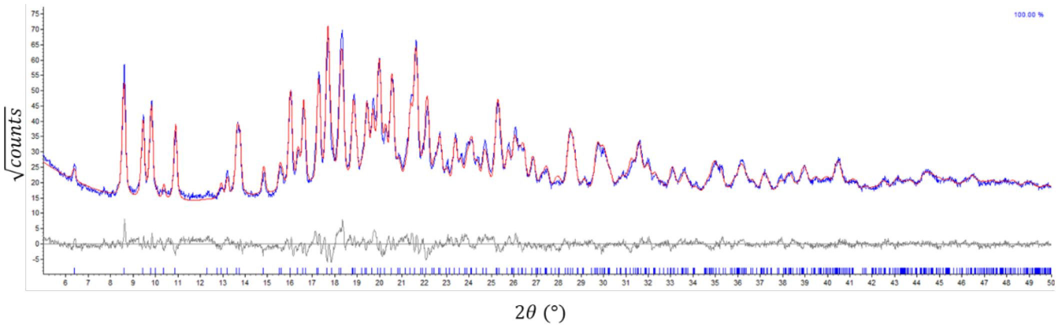

Fig. S5. Rietveld refinement of Ritonavir form II. Experimental (blue curve), calculated (red curve) and difference (grey curve) patterns of Ritonavir form II.

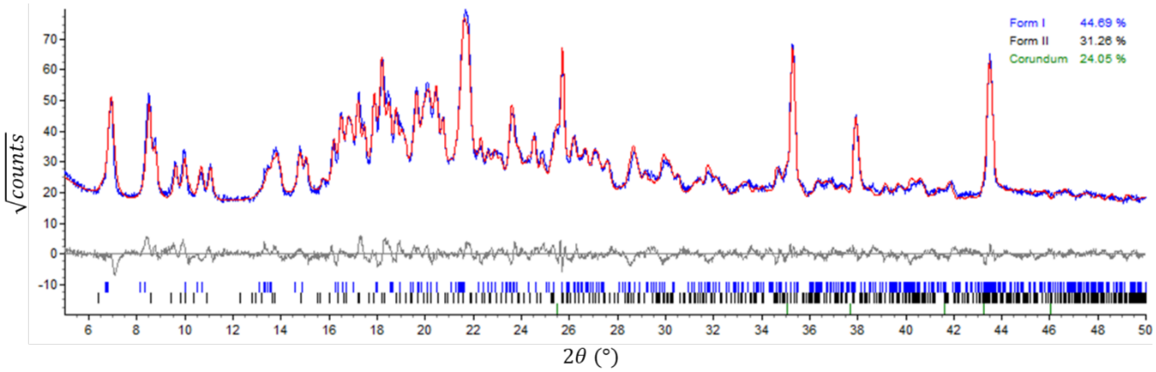

Fig. S6. Experimental (blue curve), calculated (red curve) and difference (grey curve) patterns of measurement sample A. Form-I (blue), Form-II (black) of ritonavir and corundum (green) peak marks are also indicated.

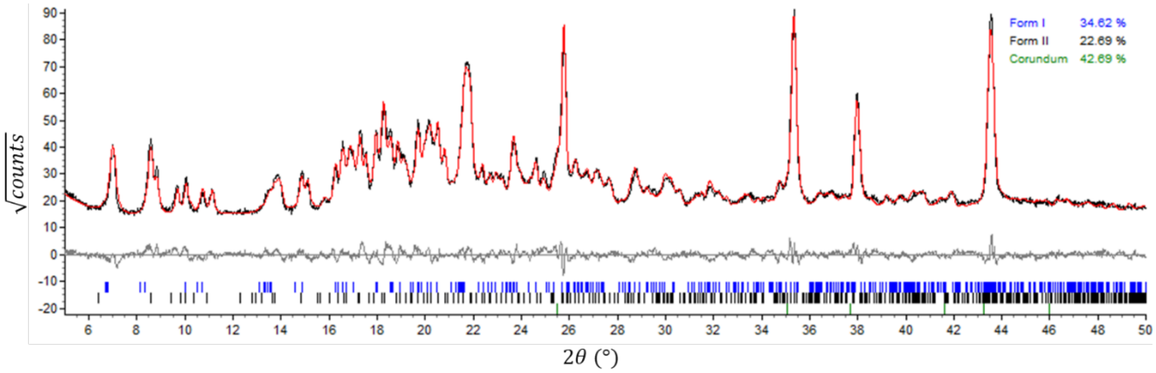

Fig. S7. Experimental (black curve), calculated (red curve) and difference (grey curve) patterns of measurement sample B. Form-I (blue), Form-II (black) of ritonavir and corundum (green) peak marks are also indicated.

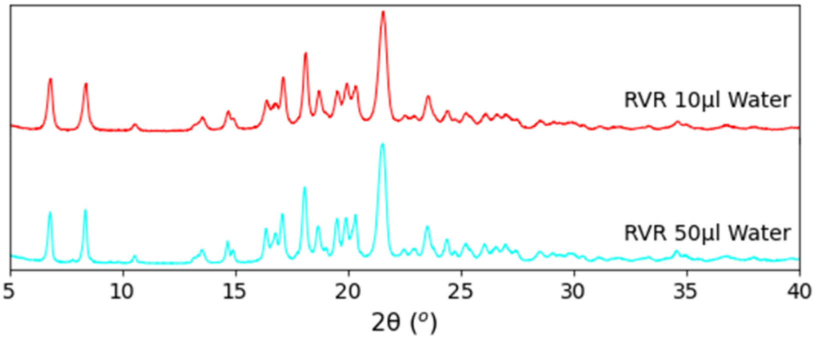

Fig. S8. The PXRD patterns of Ritonavir form I LAG ball milling with increasing volumes of Water.

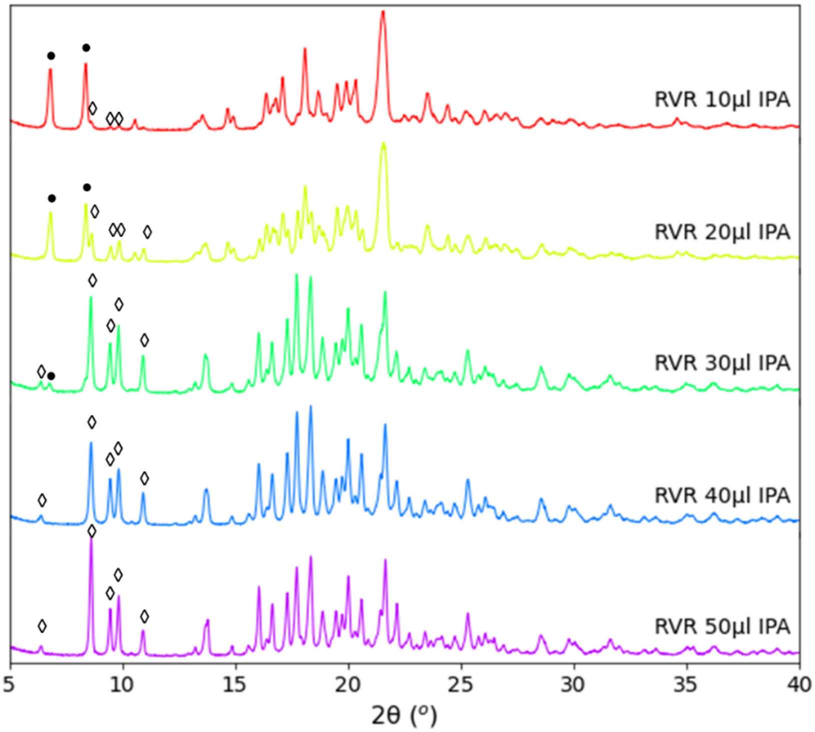

Fig. S9. The PXRD patterns of Ritonavir form I LAG ball milling with increasing volumes of IPA. Peaks relating to form-I are indicated with a circle ● and form-II with a diamond ◊.

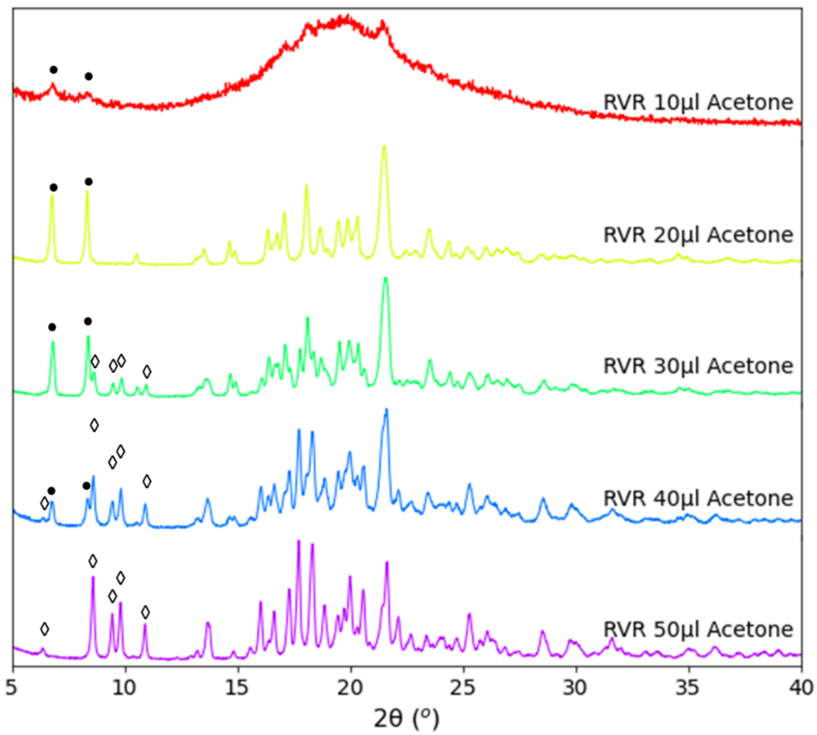

Fig. S10. The PXRD patterns of Ritonavir form I LAG ball milling with increasing volumes of Acetone. Peaks relating to form-I are indicated with a circle ● and form-II with a diamond ◊.

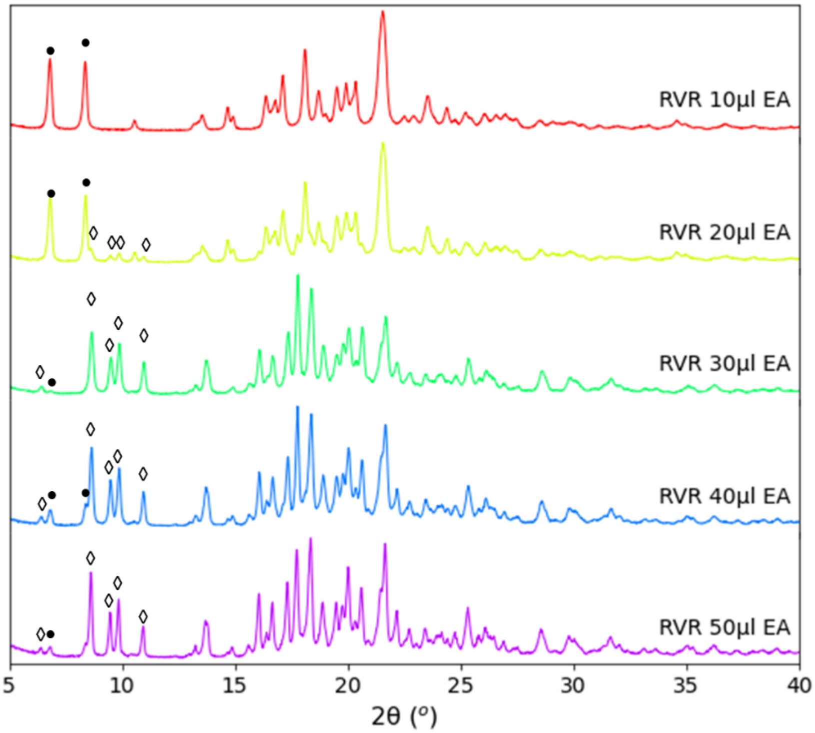

Fig. S11. The PXRD patterns of Ritonavir form I LAG ball milling with increasing volumes of EA. Peaks relating to form-I are indicated with a circle ● and form-II with a diamond ◊.

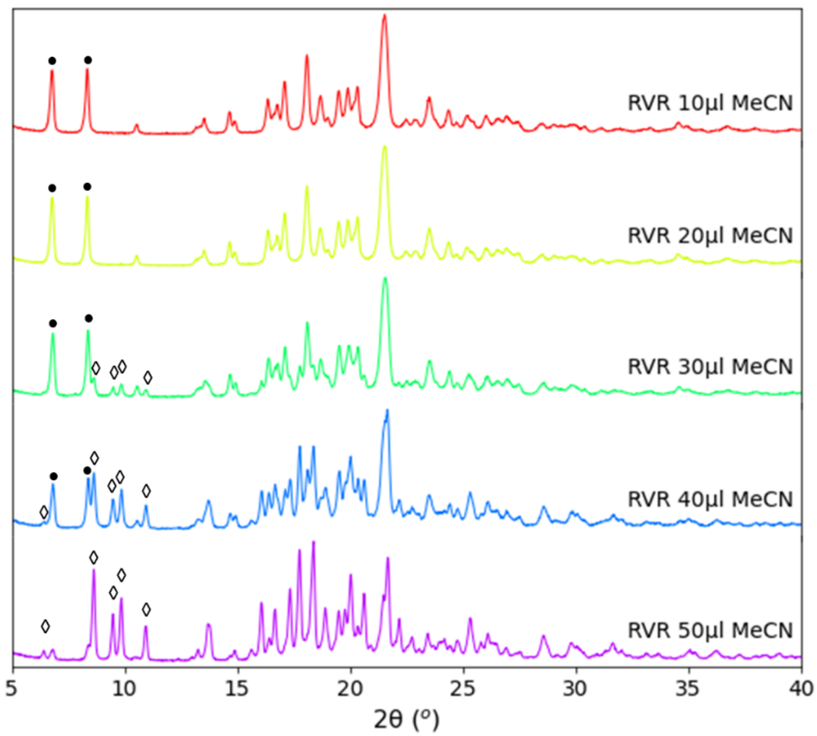

Fig. S12. The PXRD patterns of Ritonavir form I LAG ball milling with increasing volumes of MeCN. Peaks relating to form-I are indicated with a circle ● and form-II with a diamond ◊.

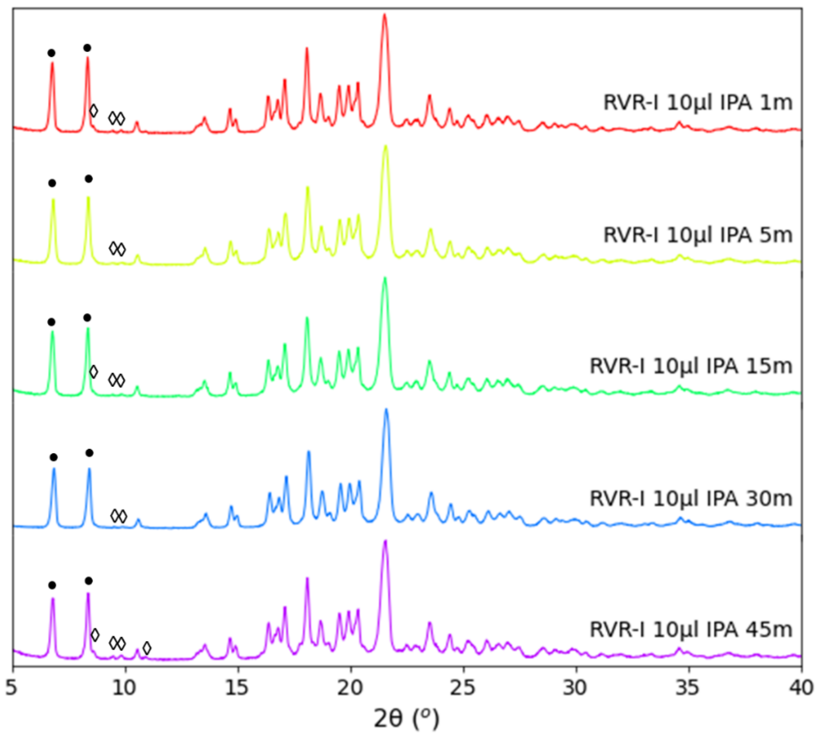

Fig. S13. The PXRD patterns of Ritonavir form **I** LAG ball milling with 10µl IPA for increasing time periods. Peaks relating to form-**I** are indicated with a circle ● and form-**II** with a diamond ◊.

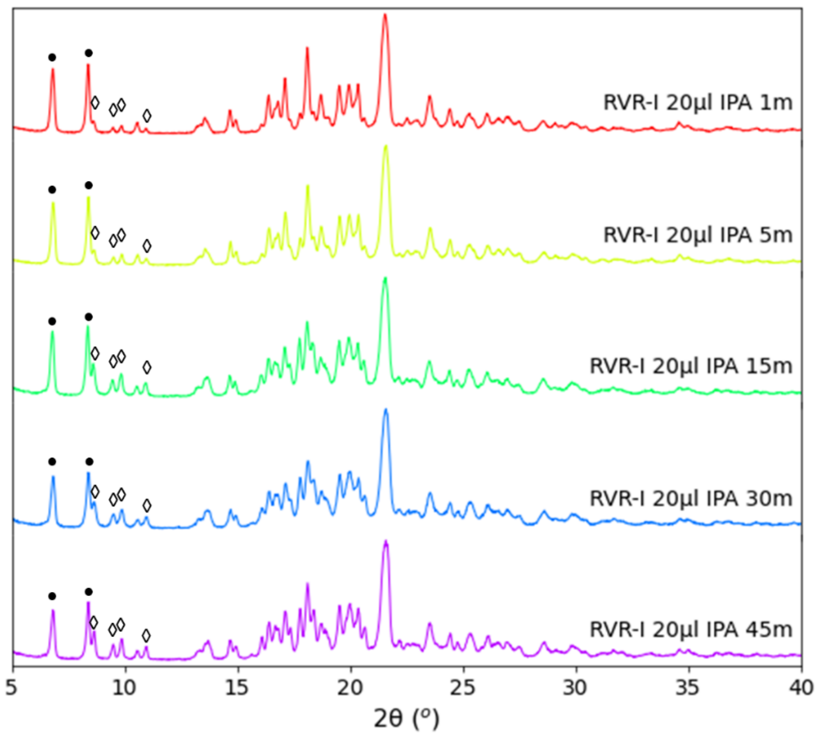

Fig. S14. The PXRD patterns of Ritonavir form I LAG ball milling with 20µl IPA for increasing time periods. Peaks relating to form-I are indicated with a circle ● and form-II with a diamond ◊.

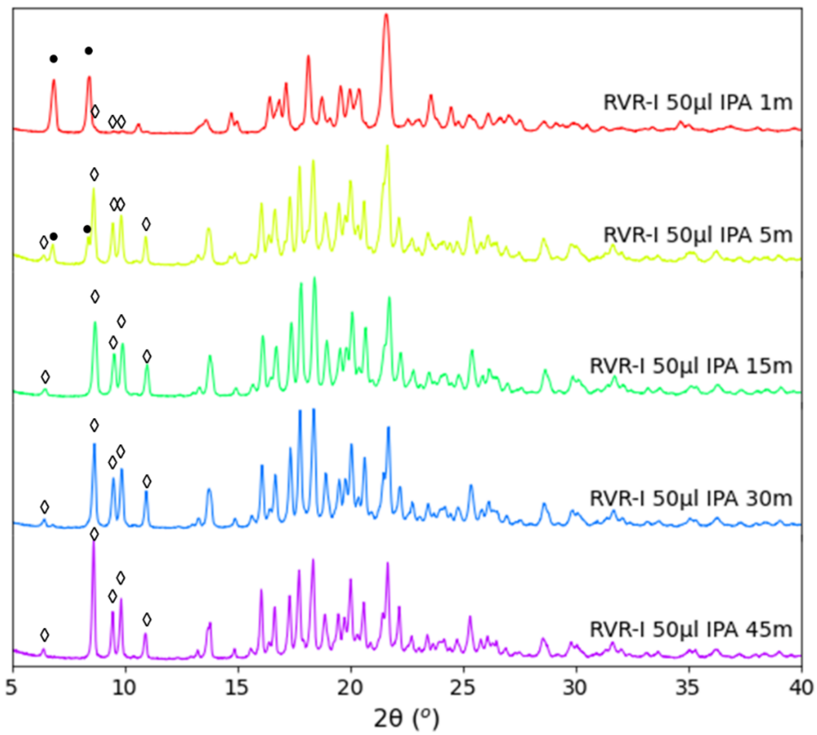

Fig. S15. The PXRD patterns of Ritonavir form I LAG ball milling with 50µl IPA for increasing time periods. Peaks relating to form-I are indicated with a circle ● and form-II with a diamond ◊.

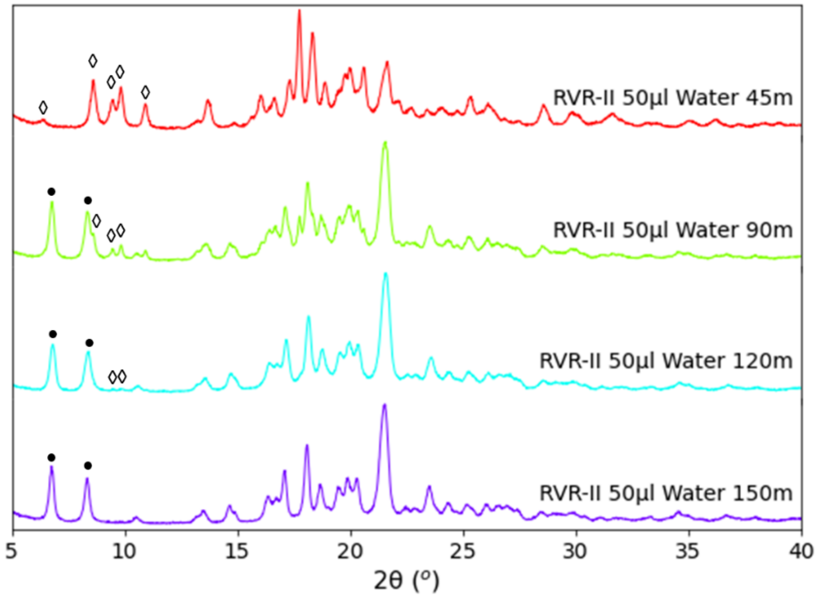

Fig. S16. The PXRD patterns of Ritonavir form **I** LAG ball milling with 50µl Water for increasing time periods. Peaks relating to form-**I** are indicated with a circle ● and form-**II** with a diamond ◊.

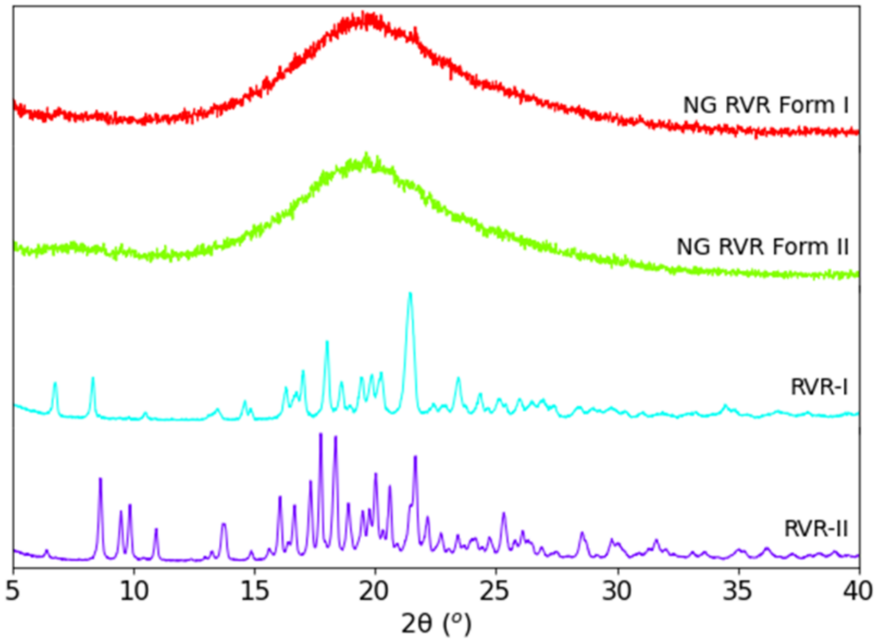

Fig. S17. Normalised PXRD patterns of the amorphous materials obtained by NG (from both polymorphic forms for 120m) and pure forms of RVR.

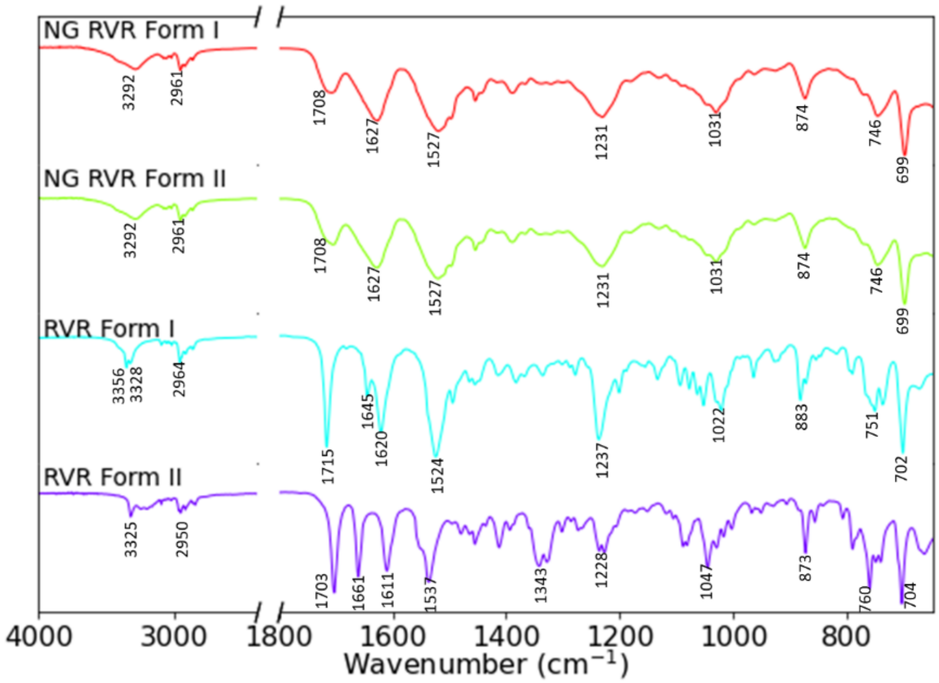

Fig. S18. FTIR spectra of the amorphous materials obtained by NG (from both polymorphic forms for 120m) and pure forms of RVR. No peaks were observed in the region between 2400 and 1800 cm^{-1}, which has been removed from the plot to improve visibility.

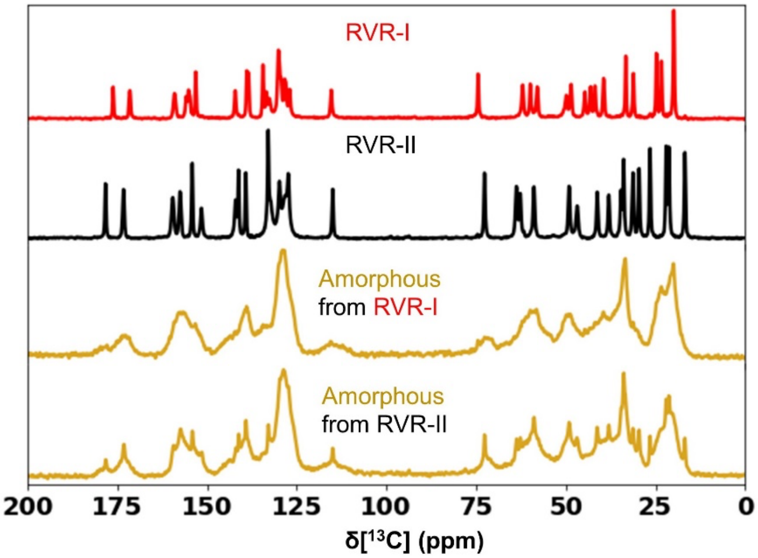

Fig. S19. ^{13}C CP/MAS of RVR Form I and RVR with their associated amorphised material.

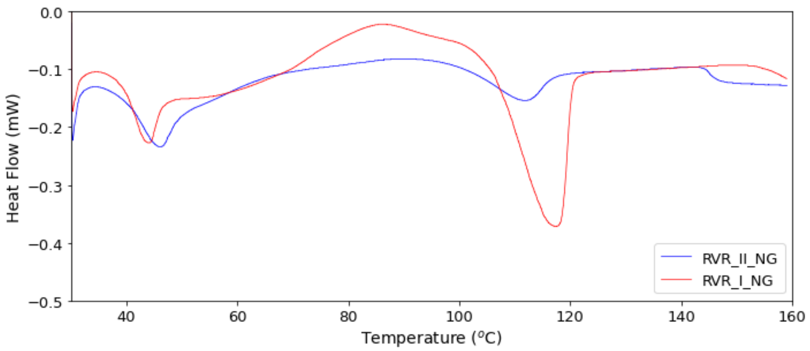

Fig. S20. DSC traces of the amorphous forms obtained by NG of RVR-I and RVR-II.

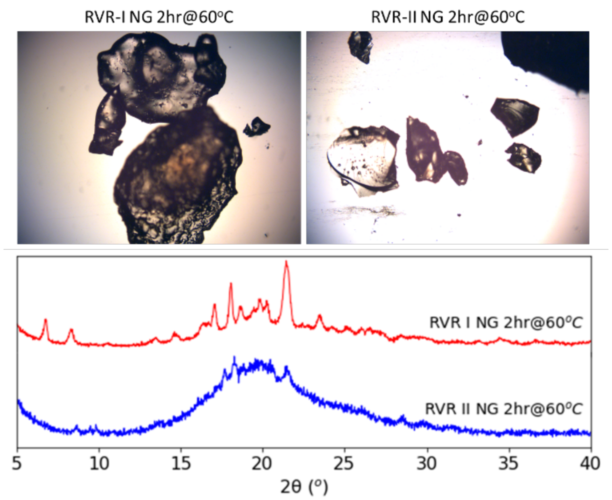

Fig. S21. Optical micrographs (top) and PXRD patterns (bottom) of the amorphous forms obtained by NG of RVR-I and RVR-II after being held at 60ºC for two hours.

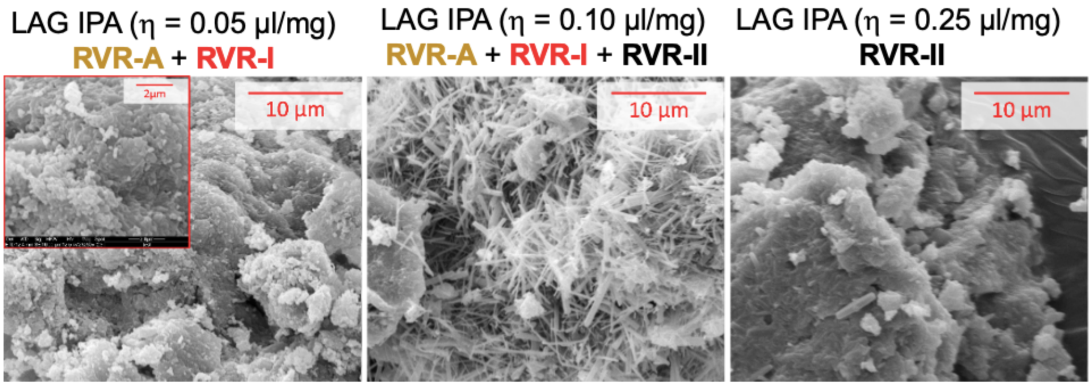

Fig. S22. SEM images of the LAG milling products with IPA at different concentrations of RVR:IPA. Milling was performed for 45 minutes.

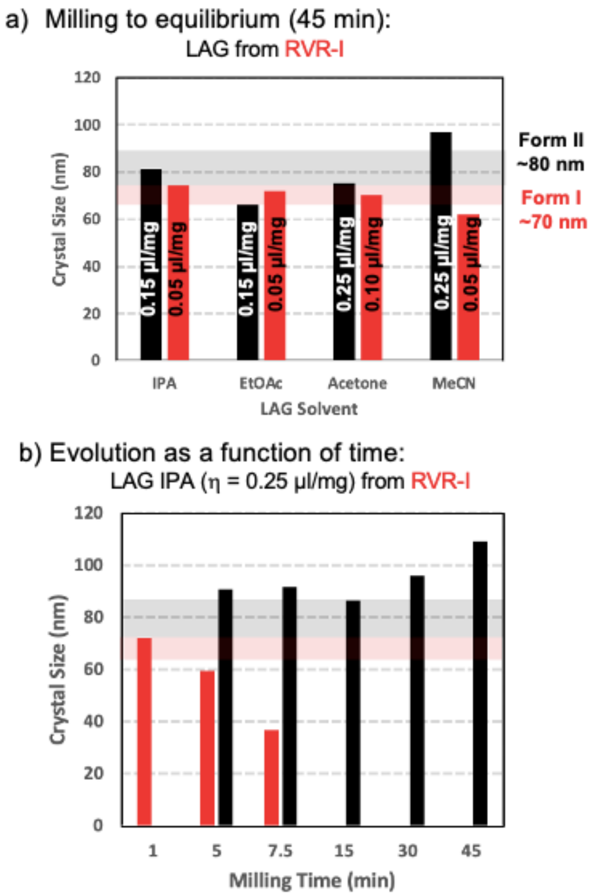

Fig. S23. Scherrer crystal size after milling equilibrium and during the kinetic experiments. All milling experiments start from Form I. a) RVR LAG for 45 min in various solvents (lowest solvent concentration affording form II -black- and highest solvent concentrations affording form I -red). b) Evolution of crystal size from form II obtained by LAG of RVR-I in IPA (0.25 µl/mg) as a function of time. At 5 min. of milling, almost 90% of RVR has converted to form II. The grey band indicates the average size for form II equilibrium size (with its standard deviation) and the red band indicates the average size for form I equilibrium size.

a) RVR-I

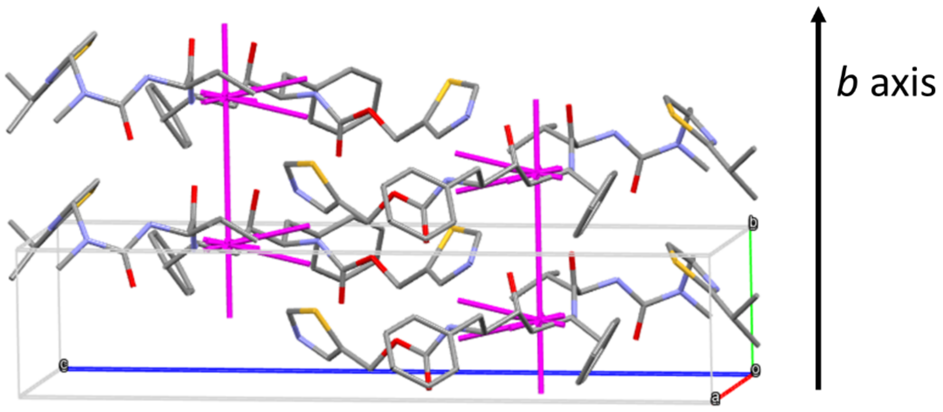

b) RVR-II

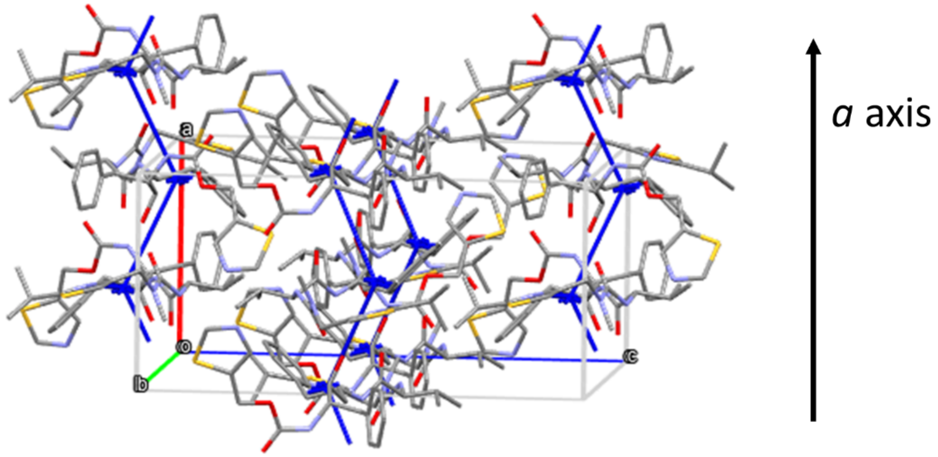

Fig. S24. Visual representations of the strongest intermolecular interactions in RVR-I (a) and RVR-II (b) obtained using the *processPIXEL* code of A. Bond. The length of each vector is proportional to the strength of the corresponding interaction relative to each polymorph.

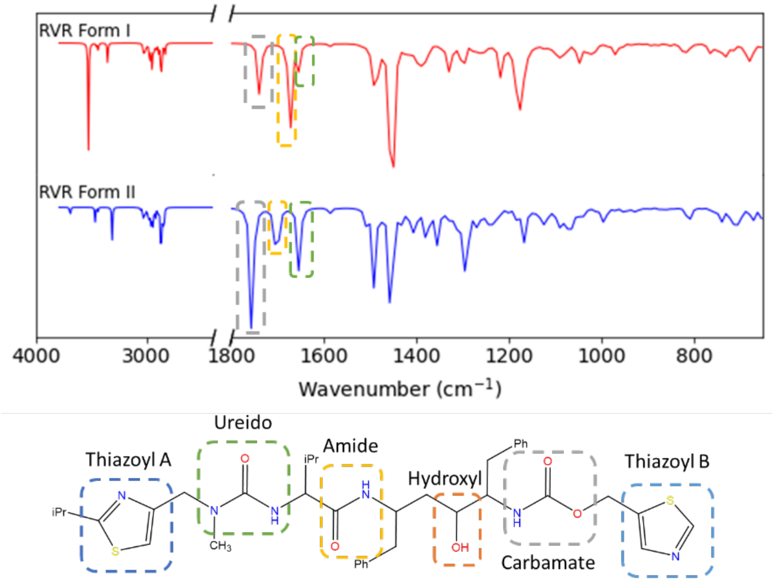

Fig. S25. Simulated IR spectra (above) of optimised conformers of the polymorphs of RVR and the chemical structure of RVR (below) with key functional groups highlighted.

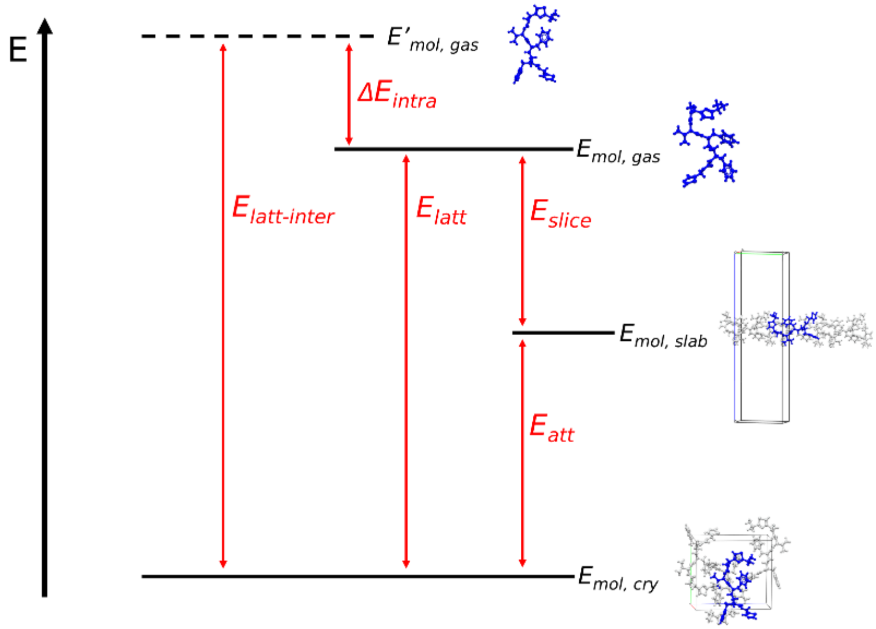

Fig. S26. Energy diagram used to define lattice, slice and attachment energies.

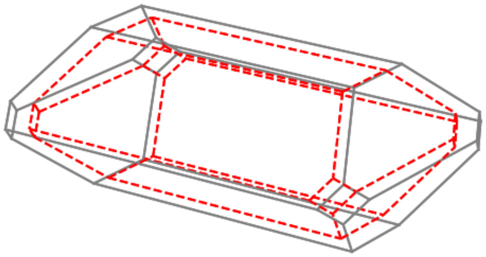

Fig. S27. A graphical representation of the method used to calculate the fraction of surface molecules in the crystal particles. An inner core (red, dashed lines) is generated by subtracting the thickness of a single (hkl) layer from the distances used to generate the particle's morphology (grey lines). The ratio of the outer shell to the total particle volume yields the total fraction of surface molecules.

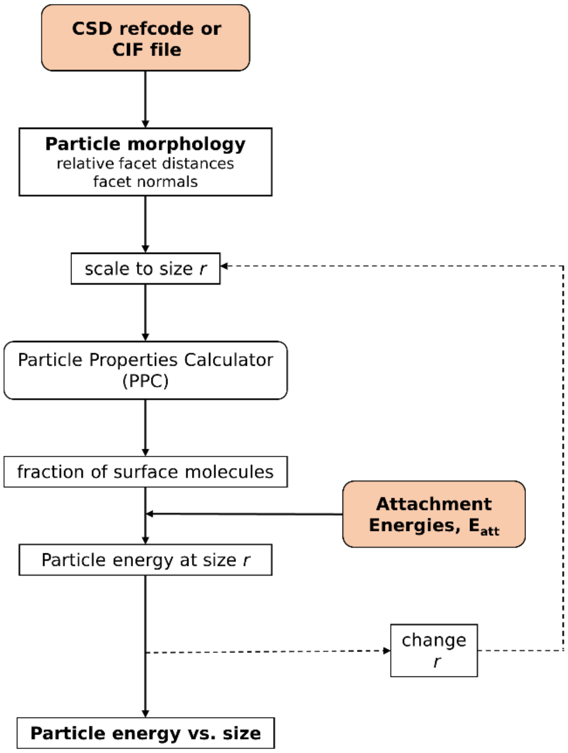

Fig. S28. A schematic view of the PEC algorithm

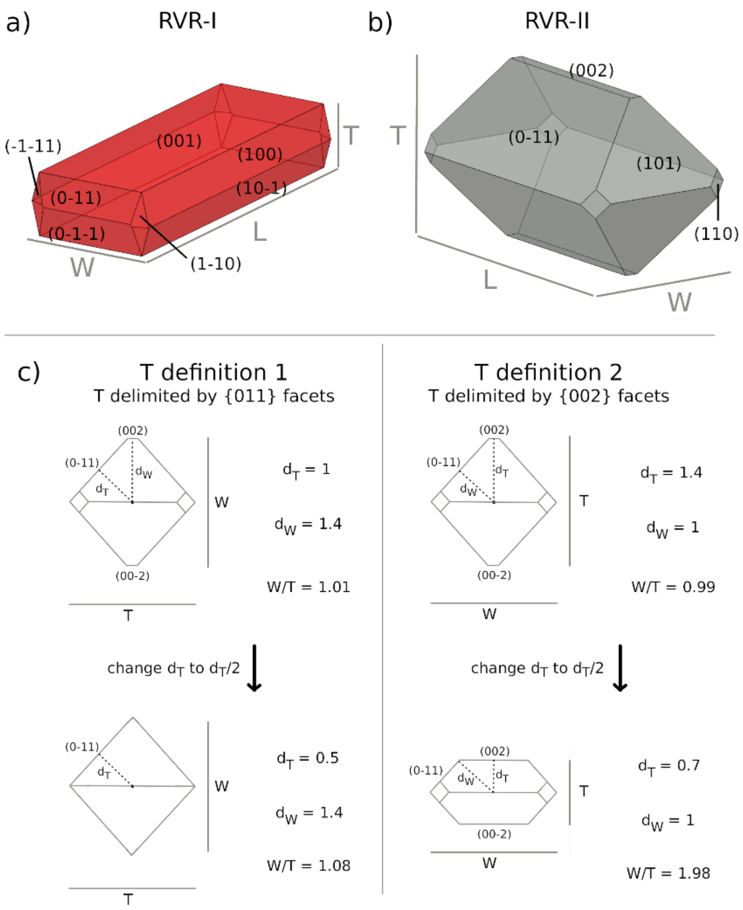

Fig. S29. Definition of the orthogonal bounding box dimensions L, W and T used to define the RVR particles' aspect ratios. (a) Attachment energy morphology of RVR-I. (b) Attachment energy morphology of RVR-II. (c) Two possible definitions of the W and T dimensions for RVR-II morphologies. In case of definition 1 (left) halving of the facet distance used to define the T dimension ($d_{(0-1-1)}$, dT) results in little change in the W/T aspect ratio. In case of definition 2 (right), instead, halving of the facet distance used to define the T dimension ($d_{(002)}$, dT) doubles the W/T aspect ratio. Definition 2 allows to define particles with a larger variety of shapes/aspect ratios.

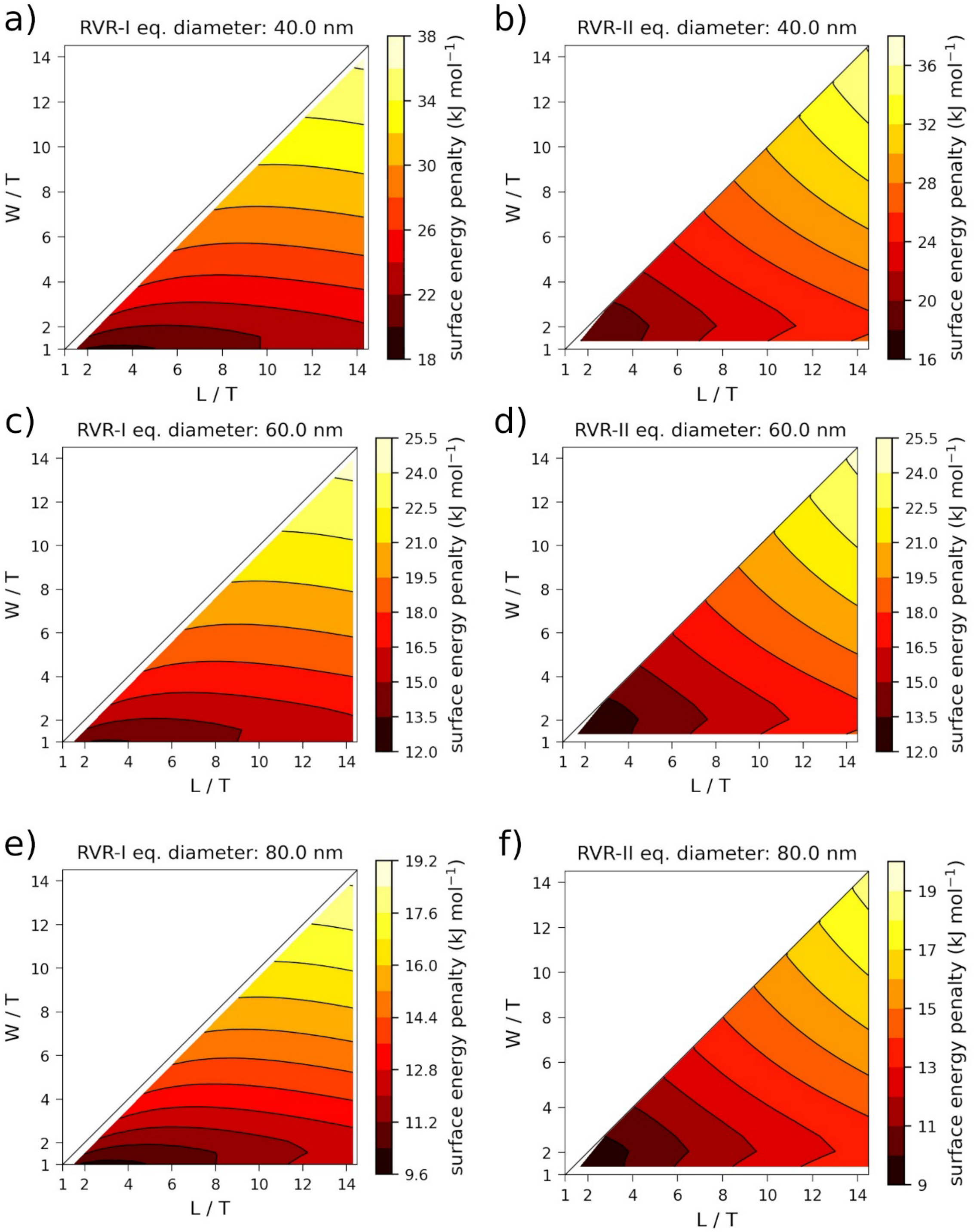

Fig. S30. Dependence of the surface energy penalty dependence on particle shape for crystal morphologies for RVR-**I** (left column) and RVR-**II** (right column) at fixed particle sizes of 40 nm (a-b), 60 nm (c-d), 80 nm (e-f). The particle size is expressed through the diameter of a sphere having equivalent volume. The particle dimensions used to define the L/T and W/T aspect ratios are visible in Supplementary Figure 29.

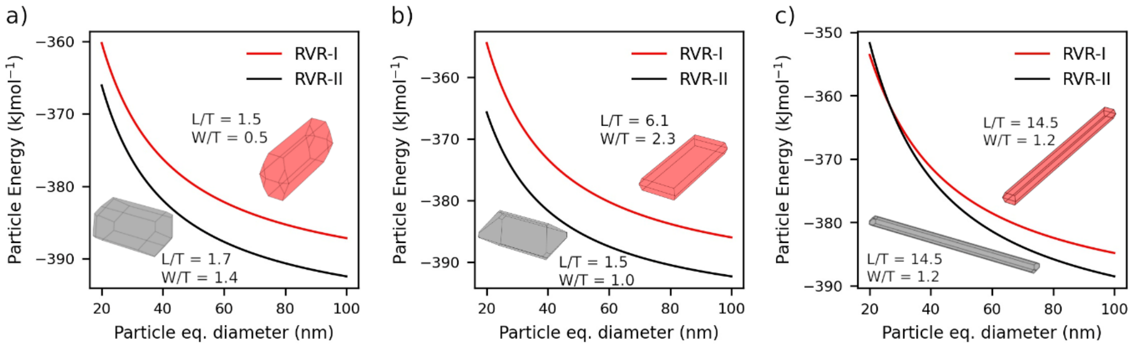

Fig. S31. Particle energy as a function of particle size (expressed as the equivalent diameter) for RVR-I and RVR-II as calculated with the PEC algorithm for four different combinations of particle morphologies. (a) morphologies corresponding to the minimum surface energy penalty; (b) attachment energy morphologies; (c) arbitrarily selected needle morphologies.

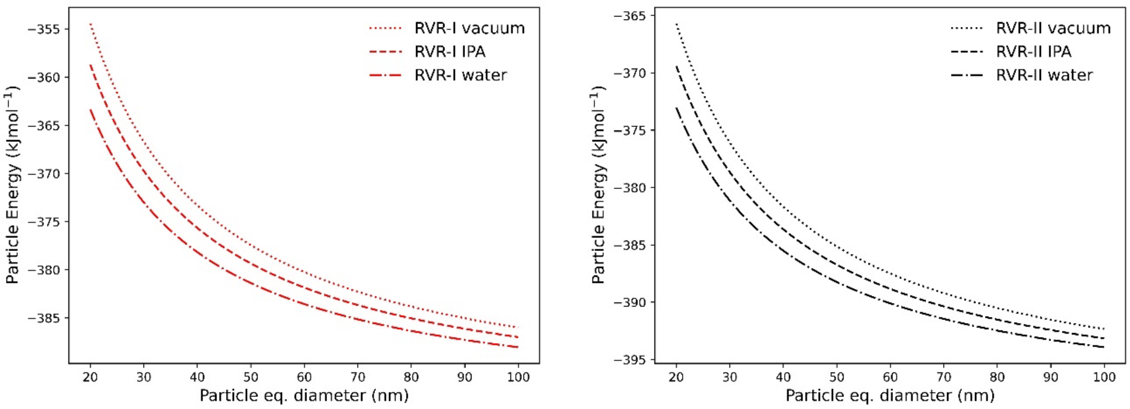

Fig. S32. Effect of the solvent on the calculated particle energies for RVR-**I** (left) and RVR-**II** (right). Energies for both plots were calculated using the respective attachment energy morphologies as input morphologies.

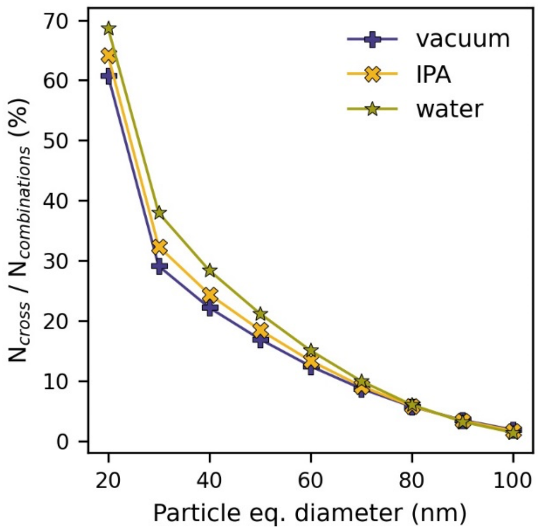

Fig. S33. Percentage of particle shapes combinations that satisfy the condition for the thermodynamic cross between RVR-I and RVR-I as a function of particle equivalent diameter. At smaller sizes, the particle energies calculated with implicit solvent models have more probability to cross compared to those calculated in vacuum.

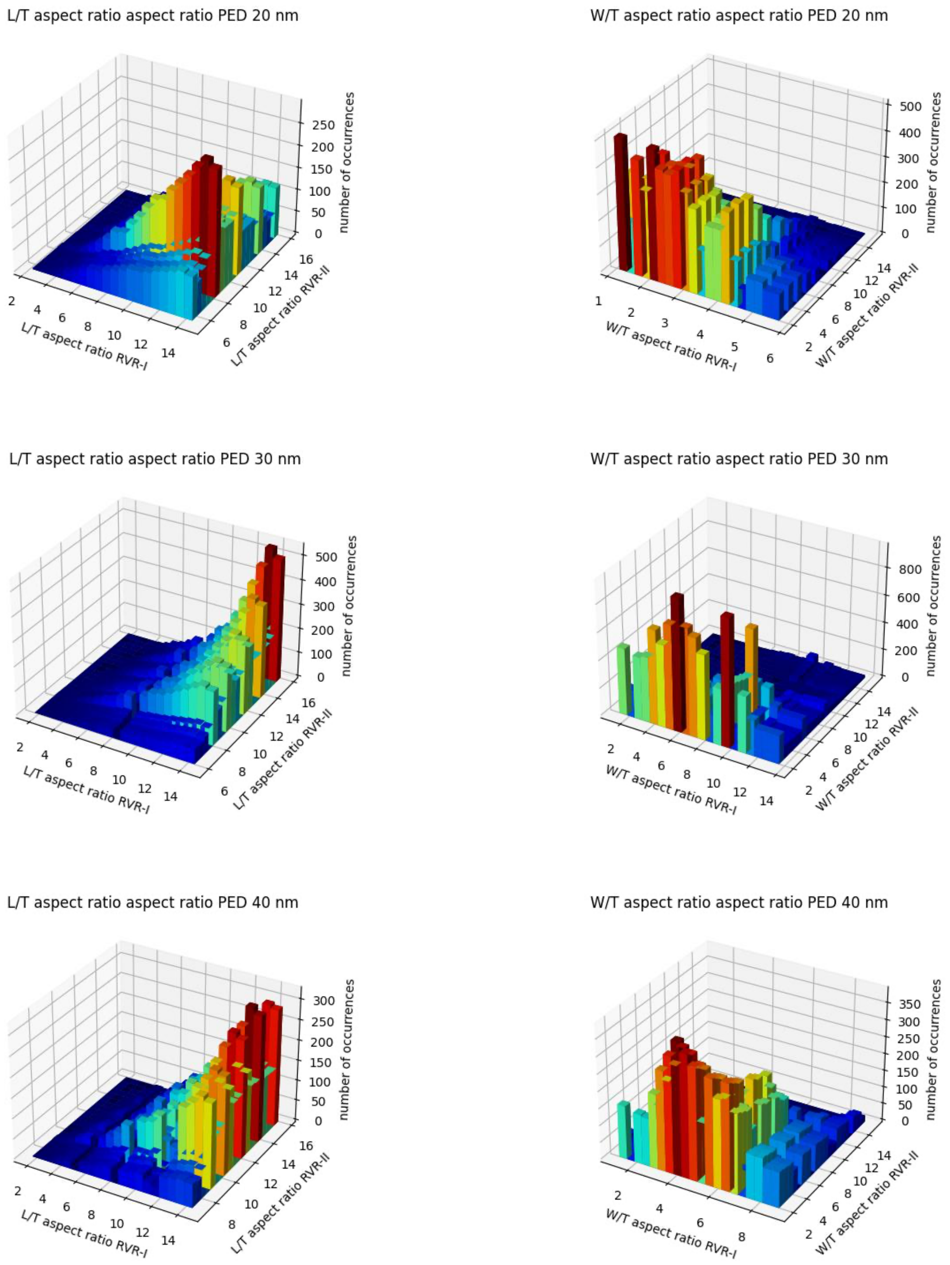

Fig. S34. Histograms showing the L/T aspect ratios (left column) and the W/T aspect ratios (right column) for morphologies of RVR-**I** and RVR-**II** affording a stability switch at PED sizes between 20 and 40 nm.

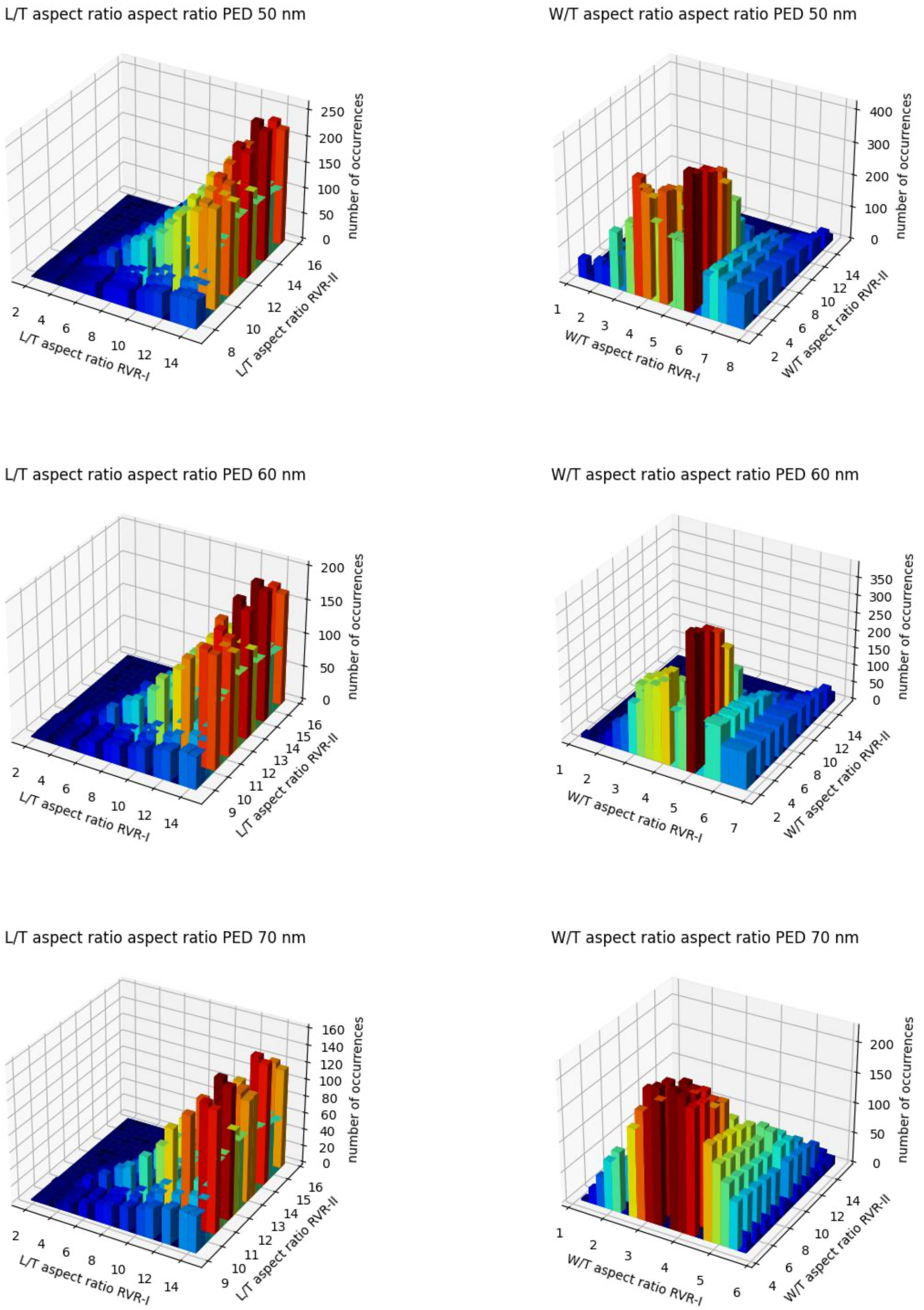

Fig. S35. Histograms showing the L/T aspect ratios (left column) and the W/T aspect ratios (right column) for morphologies of RVR-I and RVR-II affording a stability switch at PED sizes between 50 and 70 nm.

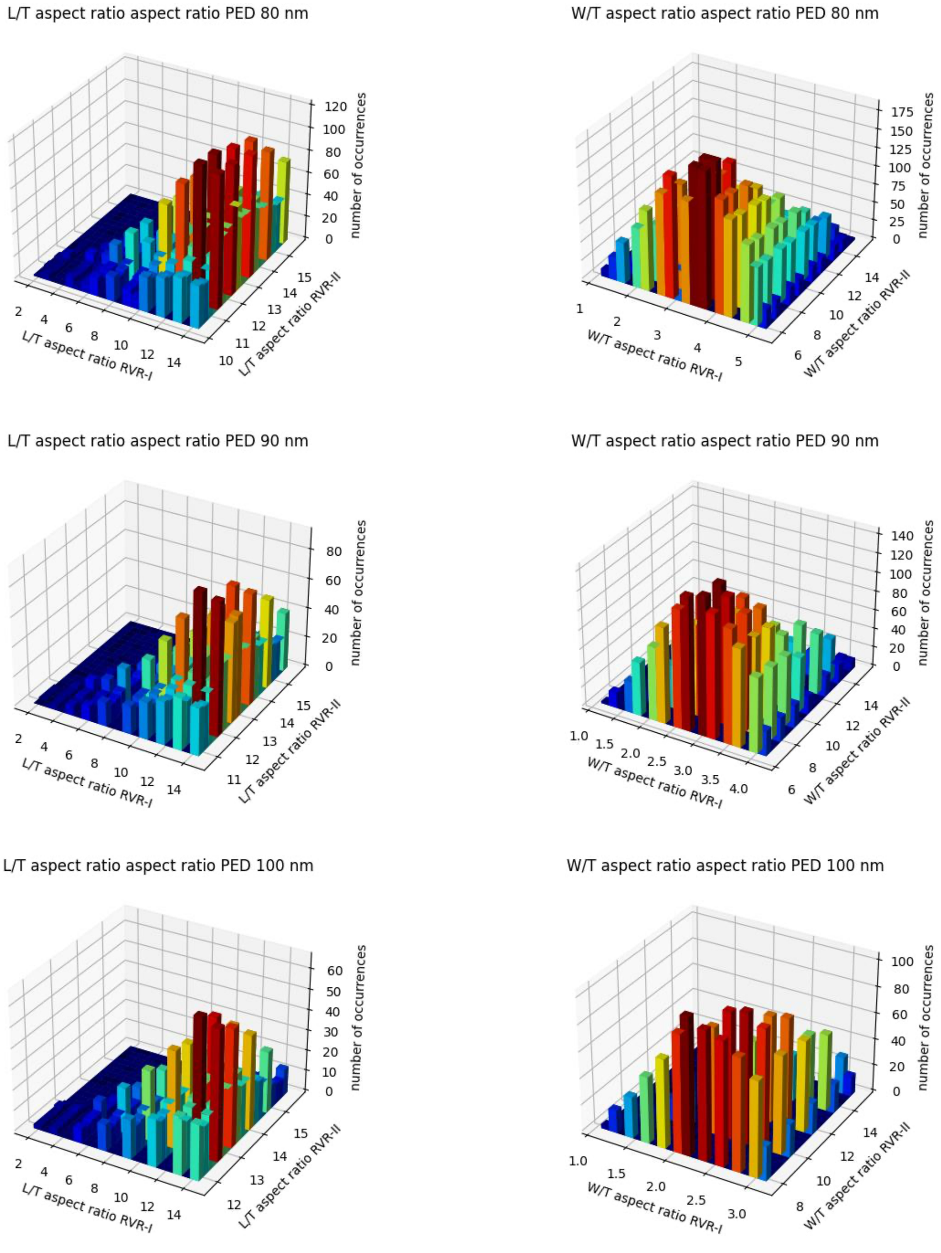

Fig. S36. Histograms showing the L/T aspect ratios (left column) and the W/T aspect ratios (right column) for morphologies of RVR-I and RVR-II affording a stability switch at PED sizes between 80 and 100 nm.

Table S1. Onset and peak temperature, melting enthalpy and enthalpy difference for the DSC melting peaks of RVR-I and RVR-II.

Form	T Onset (°C)	T peak (°C)	ΔH_{fus} (J/g)	ΔH_{fus} (kJ/mol)	ΔΔH (kJ/mol)
RVR-I	118.3	121.0	74.9	54.0	3.3
RVR-II	117.41	120.5	79.4	57.2	

Table S2. Rwp, calculated weight fractions from the refinement, experimental weight fraction of the internal standard and the estimated weight fraction of the amorphous content in the sample.

Sample	Rwp	calc %wt Form-I	calc %wt Form-II	exp %wt Al_2O_3	calc %wt Al_2O_3	sample %wt amorphous
**Sample A**	9.57	44.7%	31.3%	20.2%	24.1%	20.1%
**Sample B**	9.37	34.6%	22.7%	36.3%	42.7%	23.5%

Table S3. Contributions to the lattice energies (intermolecular) of RVR polymorphs as calculated with PIXEL. All energies in $kJmol^{-1}$

form	E_{coul}	E_{pol}	E_{disp}	E_{rep}	E_{dip}	E_{total}	ΔE
**RVR-I**	-347.0	-174.5	-494.8	640.9	-0.3	-375.7	31.5
**RVR-II**	-401.2	-198.3	-499.4	691.8	0.0	-407.2	-

Table S4. Five strongest PIXEL intermolecular interaction energies of RVR polymorphs. All energies in kJ mol^{-1}. The distance between the interacting molecules' centres of mass is also reported.

form	Symmetry[a]	Distance (Å)	E_{coul}	E_{pol}	E_{disp}	E_{rep}	E_{total}
**RVR-I**	x,-1+y,z	5.16	-175.8	-80.1	-195.5	281.5	-169.8
	1-x,0.5+y,1-z	11.77	-94.9	-53.4	-98.1	157.4	-88.9
	2-x,0.5+y,1-z	15.68	-21.4	-10.7	-48.2	51.3	-29.0
	-1+x,y,z	13.10	-21.4	-8.7	-44.0	45.2	-28.9
	1-x,-0.5+y,-z	14.49	-10.8	-6.7	-38.3	34.1	-21.7
**RVR-II**	0.5+x,1.5-y,2-z	5.35	-283.9	-132.4	-217.4	389.2	-244.5
	1-x,-0.5+y,1.5-z	11.99	-37.5	-22.1	-82.9	96.6	-45.8
	0.5-x,2-y,0.5+z	13.23	-9.9	-4.7	-30.2	22.5	-22.4
	x,-1+y,z	18.26	-32.6	-18.5	-64.2	94.4	-21.0
	0.5-x,1-y,0.5+z	14.55	-7.0	-7.2	-31.0	26.6	-18.6

[a]the symmetry operation relating the interacting molecules

Table S5. Simulated and Solid FTIR peaks for carbamate, amide and ureido carbonyl stretches. Frequencies are given in cm^{-1}.

Carbonyl Stretch	Solid-FTIR				Simulated (optimised conformers)	
	RVR-I	RVR-II	RVR-A	RVR:AcOH	RVR-I	RVR-II
Carbamate	1715	1703	1708	1706	1739	1757
Amide	1645	1661	1627 (broad)	1649	1671	1705
Ureido	1620	1611		1629	1654	1654

Table S6. Comparison of structural parameters experimental and DFT optimised for RVR-I and RVR-II.

Form	structure	Lattice parameters *(a* (Å)*, b* (Å)*, c* (Å), β (°))	rmsd-20 (Å)	rmsd-CL (Å)	rmsd-CA (°)
**RVR-I** ***P2₁***	YIGPIO02	13.344(2), 5.2150(8), 26.693(4), 103.456(2)			
	opt GD2	13.100, 5.161, 25.738, 102.981	0.386	0.570	0.274
	opt TS	13.230, 5.223, 26.191, 103.306	0.222	0.297	0.086
**Form**	**structure**	**Lattice parameters *(a* (Å)*, b* (Å)*, c* (Å))**	**rmsd-20 (Å)**	**rmsd-CL (Å)**	**rmsd-CA (°)**
**RVR-II** ***$P2_12_12_1$***	YIGPIO03	9.831(6), 18.485(11), 20.261(12)			
	opt GD2	9.548, 18.258, 19.703	0.305	0.384	-
	opt TS	9.645, 18.419, 20.049	0.159	0.167	-

Table S7. Calculated DFT energies of RVR crystal structures. All energies in kJ mol^{-1}.

	Method	RVR-I	RVR-II	ΔE (I-II)
$E_{latt\text{-}inter}$	PBE-GD2	-383.9	-423.0	39.1
	PBE-TS	-456.8	-491.2	34.4
ΔE_{intra}	PBE-GD2	57.2	82.3	-25.1
	PBE-TS	51.0	76.9	-25.9
	MP2D	61.7	91.3	-29.6
E_{latt}	PBE-GD2	-326.7	-340.7	14.0
	PBE-TS	-405.8	-414.4	8.6
	PBE-GD2 + MP2D	-322.2	-331.6	9.5
	PBE-TS + MP2D	-395.0	-399.9	4.9

Table S8. Calculated attachment energies for RVR polymorphs.

Form	Planes	M[a]	d_{hkl} / Å	**Vacuum** (ε = 1)		**IPA** (ε = 19.3)		**water** (ε = 78.4)	
				$E_{att,\{hkl\}}$ / $kJmol^{-1}$	M. I.[b]	$E_{att,\{hkl\}}$ / $kJmol^{-1}$	M. I.	$E_{att,\{hkl\}}$ / $kJmol^{-1}$	M. I.
RVR-I	{001}	2	25.49	-54.4	0.59	-50.8	0.58	-41.8	0.60
	{100}	2	12.87	-120.0	0.17	-112.7	0.18	-99.3	0.18
	{10-[illegible]	2	12.73	-121.6	0.13	-116.6	0.12	-104.2	0.10
	{011}	4	5.12	-326.5	0.11	-267.7	0.12	-230.6	0.12
	{110}	4	4.84	-368.0	0	-304.8	0	-265.1	0
	{11-[illegible]	4	4.83	-414.6	0	-341.5	0	-298.5	0
RVR-[illegible]	{011}	4	13.56	-138.0	0.72	-128.6	0.71	-113.3	0.72
	{020}	2	10.02	-188.4	0.03	-177.8	0.03	-161.6	0.01
	{101}	4	8.69	-262.3	0.23	-221.1	0.26	-195.0	0.26
	{110}	4	8.54	-318.5	0.01	-275.2	0.01	-245.7	0.01

[a] facet multiplicity; [b] morphological importance of the resulting growth morphology

Table S9. Parameters used to run the PBEM simulations presented in the main text.

	**Milling intensity, p_m [-]**	**Hold-up ratio, v [-]**	**Initial mean particle size, $\bar{y}_0$ [-]**	**Initial standard deviation, σ_0 [-].**
**Pure breakage**	2.50e-4	5.00e-2	200	1
	5.00e-4	5.00e-2	200	1
	1.00e-3	5.00e-2	200	1
	2.00e-3	5.00e-2	200	1
	4.00e-3	5.00e-2	200	1
**Pure Ostwald ripening**	0	1.25e-2	2	1
	0	2.50e-2	2	1
	0	5.00e-2	2	1
	0	1.00e-1	2	1
	0	2.00e-1	2	1
	0	1.25e-2	200	1
**Breakage + Ostwald Ripening (Effect of milling intensity)**	2.50e-4	5.00e-2	200	1
	5.00e-4	5.00e-2	200	1
	1.00e-3	5.00e-2	200	1
	2.00e-3	5.00e-2	200	1
	4.00e-3	5.00e-2	200	1
**Breakage + Ostwald Ripening (Effect of solubility)**	1.00e-3	2.00e-1	200	1
	1.00e-3	1.00e-1	200	1
	1.00e-3	5.00e-2	200	1
	1.00e-3	2.50e-2	200	1
	1.00e-3	1.25e-2	200	1

Table S10. Arbitrary simulation parameters used for our PBEM model.

**Parameter name**	**Symbol**	**Value**
Breakage limit	k_b [m]	1
-	x_{ref} [m]	1e-6
Shape factor	k_v [-]	π/6
Density	ρ [kg m^{-3}]	2000
Reference length	x_0 [m]	1000
Scaled capillary length	a^* [-]	0.01
Growth rate constant	k [kg m^{-3} s^{-1}]	0.5
-	β [-]	0
-	λ [-]	1

SI References

1. S. E. Wright, M. J. Bryant, A. J. Cruz-Cabeza, Is it usual to be unusual? An investigation into molecular conformations in organic crystals. *CrystEngComm* **22**, 7217–7228 (2020).

2. D. Chakraborty, N. Sengupta, D. J. Wales, Conformational Energy Landscape of the Ritonavir Molecule. *J. Phys. Chem. B* **120**, 4331–4340 (2016).

3. J. S. O. Evans, Advanced Input Files & Parametric Quantitative Analysis Using Topas. *Materials Science Forum* **651**, 1–9 (2010).

4. A. A. Coelho, TOPAS and TOPAS-Academic: an optimization program integrating computer algebra and crystallographic objects written in C++. *Journal of Applied Crystallography* **51**, 210–218 (2018).

5. C. R. Groom, I. J. Bruno, M. P. Lightfoot, S. C. Ward, The Cambridge Structural Database. *Acta Crystallographica Section B* **72**, 171–179 (2016).

6. A. M. Belenguer, *et al.*, Understanding the Influence of Surface Solvation and Structure on Polymorph Stability: A Combined Mechanochemical and Theoretical Approach. *J. Am. Chem. Soc.* **140**, 17051–17059 (2018).

7. A. Gavezzotti, Efficient computer modeling of organic materials. The atom–atom, Coulomb–London–Pauli (AA-CLP) model for intermolecular electrostatic-polarization, dispersion and repulsion energies. *New J. Chem.* **35**, 1360–1368 (2011).

8. A. Gavezzotti, L. L. Presti, S. Rizzato, Molecular dynamics simulation of organic materials: structure, potentials and the MiCMoS computer platform. *CrystEngComm* **24**, 922–930 (2022).

9. M. J. Frisch, *et al.*, Gaussian16 Revision C.01 (2016).

10. A. D. Bond, *processPIXEL* : a program to generate energy-vector models from Gavezzotti's *PIXEL* calculations. *J Appl Crystallogr* **47**, 1777–1780 (2014).

11. I. M. Alecu, J. Zheng, Y. Zhao, D. G. Truhlar, Computational Thermochemistry: Scale Factor Databases and Scale Factors for Vibrational Frequencies Obtained from Electronic Model Chemistries. *J. Chem. Theory Comput.* **6**, 2872–2887 (2010).

12. S. Pinchas, D. Ben-Ishai, The Carbonyl Absorption of Carbamates and 2-Oxazolidones in the Infrared Region. *J. Am. Chem. Soc.* **79**, 4099–4104 (1957).

13. P. Hartman, P. Bennema, The attachment energy as a habit controlling factor: I. Theoretical considerations. *Journal of Crystal Growth* **49**, 145–156 (1980).

14. S. Grimme, Semiempirical GGA-type density functional constructed with a long-range dispersion correction. *Journal of Computational Chemistry* **27**, 1787–1799 (2006).

15. A. Tkatchenko, M. Scheffler, Accurate Molecular Van Der Waals Interactions from Ground-State Electron Density and Free-Atom Reference Data. *Phys. Rev. Lett.* **102**, 073005 (2009).

16. P. Sacchi, M. Lusi, A. J. Cruz-Cabeza, E. Nauha, J. Bernstein, Same or different – that is the question: identification of crystal forms from crystal structure data. *CrystEngComm* **22**, 7170–7185 (2020).

17. G. J. O. Beran, S. E. Wright, C. Greenwell, A. J. Cruz-Cabeza, The interplay of intra- and intermolecular errors in modeling conformational polymorphs. *The Journal of Chemical Physics* **156**, 104112 (2022).

18. S. R. Chemburkar, *et al.*, Dealing with the Impact of Ritonavir Polymorphs on the Late Stages of Bulk Drug Process Development. *Org. Process Res. Dev.* **4**, 413–417 (2000).

19. J. D. H. Donnay, D. Harker, A new law of crystal morphology extending the Law of Bravais. *American Mineralogist* **22**, 446–467 (1937).

20. K. Mathew, R. Sundararaman, K. Letchworth-Weaver, T. A. Arias, R. G. Hennig, Implicit solvation model for density-functional study of nanocrystal surfaces and reaction pathways. *J. Chem. Phys.* **140**, 084106 (2014).

21. K. Mathew, V. S. C. Kolluru, S. Mula, S. N. Steinmann, R. G. Hennig, Implicit self-consistent electrolyte model in plane-wave density-functional theory. *J. Chem. Phys.* **151**, 234101 (2019).

22. F. Lai, Y. Xie, Y. Chen, H. Guo, Numerical Method for Calculating Nanocrystals' Edge Energies from Experimentally Observed Shape Evolution. *J. Phys. Chem. C* **124**, 3835–3842 (2020).

23. M. Iggland, M. Mazzotti, Population Balance Modeling with Size-Dependent Solubility: Ostwald Ripening. *Crystal Growth & Design* **12**, 1489–1500 (2012).

24. L. Bosetti, M. Mazzotti, Population Balance Modeling of Growth and Secondary Nucleation by Attrition and Ripening. *Crystal Growth & Design* **20**, 307–319 (2020).

25. R. J. LeVeque, *Finite Volume Methods for Hyperbolic Problems* (Cambridge University Press, 2002) https:/doi.org/10.1017/CBO9780511791253.

26. S. Qamar, M. P. Elsner, I. A. Angelov, G. Warnecke, A. Seidel-Morgenstern, A comparative study of high resolution schemes for solving population balances in crystallization. *Computers & Chemical Engineering* **30**, 1119–1131 (2006).

27. R. Kumar, J. Kumar, Numerical simulation and convergence analysis of a finite volume scheme for solving general breakage population balance equations. *Applied Mathematics and Computation* **219**, 5140–5151 (2013).
